# A single-building damage detection model based on multi-feature fusion: A case study in Yangbi

**DOI:** 10.1016/j.isci.2023.108586

**Published:** 2023-11-29

**Authors:** Haoguo Du, Xuchuan Lin, Jinzhong Jiang, Yongkun Lu, Haobiao Du, Fanghao Zhang, Fengyan Yu, Tao Feng, Xiaofang Wu, Guanling Peng, Shurong Deng, Shifang He, Xianfu Bai

**Affiliations:** 1 Yunnan Earthquake Agency, Kunming 650224, China; 2 Institute of Engineering Mechanics, China Earthquake Administration, Harbin 150080, China; 3 Arm Engineering University of PLA, Nanjing 210000, China

**Keywords:** Remote sensing, Space sciences, Engineering

## Abstract

Accurate and effective identification, determination of the location, and classification of damaged buildings are essential after destructive earthquakes. However, the accuracy of image change detection is limited because of the many texture features and changes in non-building information. In this context, a model for single-building damage detection based on multi-feature fusion is proposed. First, the normalized Digital Surface Model (nDSM) was extracted from the DSM through iterative filtering and point cloud thinning, followed by the extraction of building contour information. Next, single-building images were generated from different data sources through the region of interest (ROI), and the optimal texture feature parameters were extracted for fusion. Afterward, principal-component analysis (PCA) was conducted to suppress multi-feature correlation-induced information redundancy. Finally, the damage to buildings was quantitatively evaluated, and the model was compared with 13 models. The results confirmed the practicability of the model for the Yangbi *M*_*S*_6.4 and Honghe *M*_*S*_5.0 earthquakes.

## Highlights

• Normalized Digital Surface Model (nDSM) improves the recognition accuracy
• The model eliminates the effects of changes in non-building information
• Multi-feature fusion is used to improve the detection accuracy
• This model realizes earthquake damage identification of single building

## Graphical abstract

**Figure:**
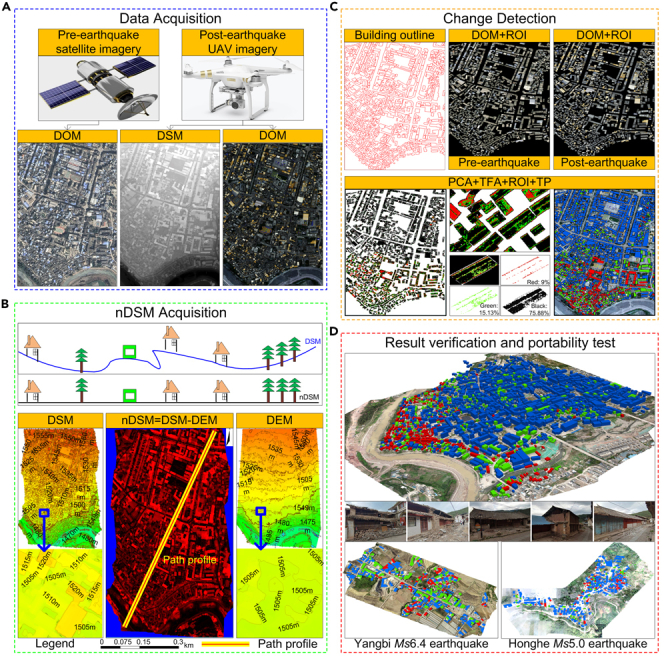

## Introduction

More than 50% of China’s cities, including about 70% of large- and medium-sized cities, are located in areas with intensity of 7° and above, exposing China to one of the highest country-scale earthquake disaster risks in the world.^1^ Following an earthquake, a timely understanding of the disaster situation is essential for carrying out loss assessment and disaster emergency response.^2^ Information is collected as a combination of an unmanned aerial vehicle’s (UAV) digital orthophoto map (DOM), DSM image, and satellite images, which can be used to identify, locate, and classify damaged buildings.

In general, there are 3 main methods for detecting post-earthquake damaged buildings via remote sensing images: visual interpretation, single-temporal detection via DOM images by machine learning, and dual-temporal detection in DOM images by machine learning. Among them, visual interpretation is currently the most accurate method for damage detection. It depends on field investigation on the ground and requires investigators to assess both the inside and outside of buildings. As a detailed assessment, the visual interpretation is accurate; however, it relies heavily on manual annotation, which is laborious, thus undermining the urgency after earthquakes.^3^^,^^4^^,^^5^^,^^6^^,^^7^ Therefore, it is very important to construct an automatic post-earthquake damage detection model.

To enhance detection accuracy and efficiency, massive machine learning algorithms have been introduced.^8^^,^^9^^,^^10^^,^^11^ For instance, Song et al.^12^ took the Wenchuan earthquake as an example; proposed an intelligent evaluation method based on deep learning, super pixel segmentation, and mathematical morphology; and used the model to evaluate the destruction degree of buildings damaged by an earthquake. They found that image segmentation is very tedious, trivial, and time-consuming, and they had to randomly select a certain number of samples from the images. Thus, the results were easily influenced by their subjectivity. Ma et al.^13^ proposed a modified convolutional neural network (CNN) Inception v3 architecture and used the Yushu earthquake as an example to address the problems of unsatisfactory image segmentation and difficult feature selection in object-oriented methods. This architecture automatically selects the best features, thus solving the problem of difficult feature selection. The results showed that the limited sample size for training in CNN and the confusion between collapsed buildings and exposed ground led to classification errors for buildings. Miura et al.^14^ proposed deep-learning-based recognition of collapsed, standing, and blue-tarpaulin-covered buildings on post-disaster aerial images based on aerial images obtained from the Kumamoto and Kobe earthquakes in Japan. The damaged buildings covered with blue tarpaulin were identified. During the test, it was difficult to identify the collapse of rubble and soft layers of buildings from the images due to insufficient sample training. By taking the Yangbi *M*_*S*_6.4 earthquake in Yunnan, China, as an example, Jing et al.^15^ proposed a neural network model based on YOLOv5s-ViT-BiFPN for a detection model for rural houses. Their classification results for different regions were quite different due to the application of various platforms and sensors after earthquakes and the differences in the types and scales of remote sensing data. Although the studies mentioned earlier have made progress, some have limitations. The sample size used for post-earthquake image training is limited, the training is easily affected by experimenters, and the system is prone to classification errors.

To address the problem of insufficient training samples, Rashidian et al.^16^ proposed the use of high-resolution RGB satellite images and a modified U-Net CNN to detect demolished buildings after natural disasters based on the existing xView ready-to-use dataset in the United States. They used the *M*_*S*_7.1 earthquake in central Mexico as an example. Yang et al.^17^ constructed a sample dataset of damaged buildings based on many disaster images retrieved from the existing xBD dataset in the United States. Moreover, taking the Wenchuan earthquake in 2008 as an example, they proposed the portability of the CNN model for identifying damaged buildings caused by earthquakes. Although the accuracy and efficiency of damaged building identification improved, the portability of the model was low, and the results were affected by inaccurate building boundaries, which were attributed to differences between the sample dataset sensor and test data sensor.^18^

To avoid the difference between the existing sample dataset and test data and the problem of inaccurate building boundaries, Qing et al.^19^ proposed a hierarchical building damage assessment workflow based on CNN super-pixel direct remote sensing dual-phase change detection. They used the Ludian earthquake as an example to solve the problems of low efficiency and inaccurate building boundaries caused by using single post-earthquake images alone. During the test, it was necessary to establish a large disaster remote sensing dataset to improve the portability of the CNN model. Moreover, there was considerable error in the results due to the influence of non-building changes and massive image features. However, with the introduction of a gray-level co-occurrence matrix (GLCM), texture features were proven conducive to the detection of damaged buildings. The abovementioned problems were solved by the dual-temporal change detection method, which assesses the difference between pre-earthquake and post-earthquake images to extract the contour of damaged buildings.^20^^,^^21^^,^^22^^,^^23^^,^^24^^,^^25^^,^^26^^,^^27^

Information on damaged buildings can be acquired through the abovementioned research. Furthermore, the dual-temporal change detection method can effectively avoid the error caused by insufficient training samples, and it performs better than the single-temporal change detection method in recognition, especially in the case of introducing image texture features. However, some errors in the change detection results occur due to the influence of non-building changes and the existence of many texture feature parameters.^28^^,^^29^^,^^30^^,^^31^ To address these two problems, (1) DSM images have been introduced to remove non-building information and highlight building boundary information. Ge et al.,^32^ for example, found remarkable changes in the height and space of buildings in earthquake-stricken areas. Wang and Li^33^ used the Port-Au-Prince earthquake in Haiti as an example and adopted normalized DSM (nDSM) to eliminate the effects of vegetation, terrain, and shadows, to prevent confusion between damaged buildings and non-buildings. Moreover, they proposed a novel multi-stage urban building damage extraction method. The extraction accuracy could be maximized only by using optimal thresholds of NDVI, nDSM, and brightness, which is in agreement with previous findings. In terms of post-earthquake height change of buildings, Tong et al.^34^ used the Wenchuan earthquake as an example and calculated the difference between pre-earthquake and post-earthquake digital elevation models (DEM) regenerated from pre-earthquake and post-earthquake stereoscopic images to determine the area of collapsed buildings. They found that DEM data do not have an accurate pixel response to the damage to buildings. This was in contrast to optical data, and there was no significant difference between the change in the threshold of building damage percentage and the degree of building damage in polygons. (2) An optimal texture feature combination is determined to avoid feature redundancy.^35^^,^^36^ For example, a correlation change detection method was proposed by Li et al.^37^ based on changes to the principal components of the texture feature set, as the sheer number of parameters makes the selection difficult to optimize. The fusion of multi-texture information is realized. This method avoids the redundancy among features while still making full use of them to realize the detection of damaged buildings. Although both methods have achieved good results in detecting changes in damaged buildings, they are not accurate enough to detect individual damaged buildings and are affected by insufficient image resolution. (3) Given the lack of access to 3D data in some areas of the world, building height is one of the most difficult attributes to obtain. One of the most widely used methods to calculate building height is based on the shadows that appear in images.^38^^,^^39^ This method is still used today, despite its limitations: it cannot be used in areas with high construction density, obstructions, or sloping terrain. One alternative to analyzing images captured by passive sensors is using three-dimensional data taken by active sensors such as SAR or LiDAR.^40^ SAR data are becoming increasingly accessible and offer high resolutions, with ground sampling distances (GSDs) of up to 1 m, but there are limitations: long data acquisition time and short update time.^41^ LiDAR point clouds are even more accurate, providing densities of several points per square meter, but there are limitations: LiDAR acquisition of ground object information is insufficient, and DSM data types are single.^42^^,^^43^ Another approach is to use stereoscopic images, which are based on the DSM dense point cloud obtained from the sparse point cloud using the multi-view stereo reconstruction method. The method has the following advantages: (1) the problems of insecurity, long acquisition time, and the short update in collecting field DSM data manually are avoided; (2) the problems of a lack of information about ground objects and the single type of DSM data obtained using LiDAR are addressed; (3) data matching accuracy is high because DOM and DSM data are acquired by the same sensors. Therefore, in this study, the author used the DSM image derived from the drone DOM image to eliminate non-building information.

**Figure 1:**
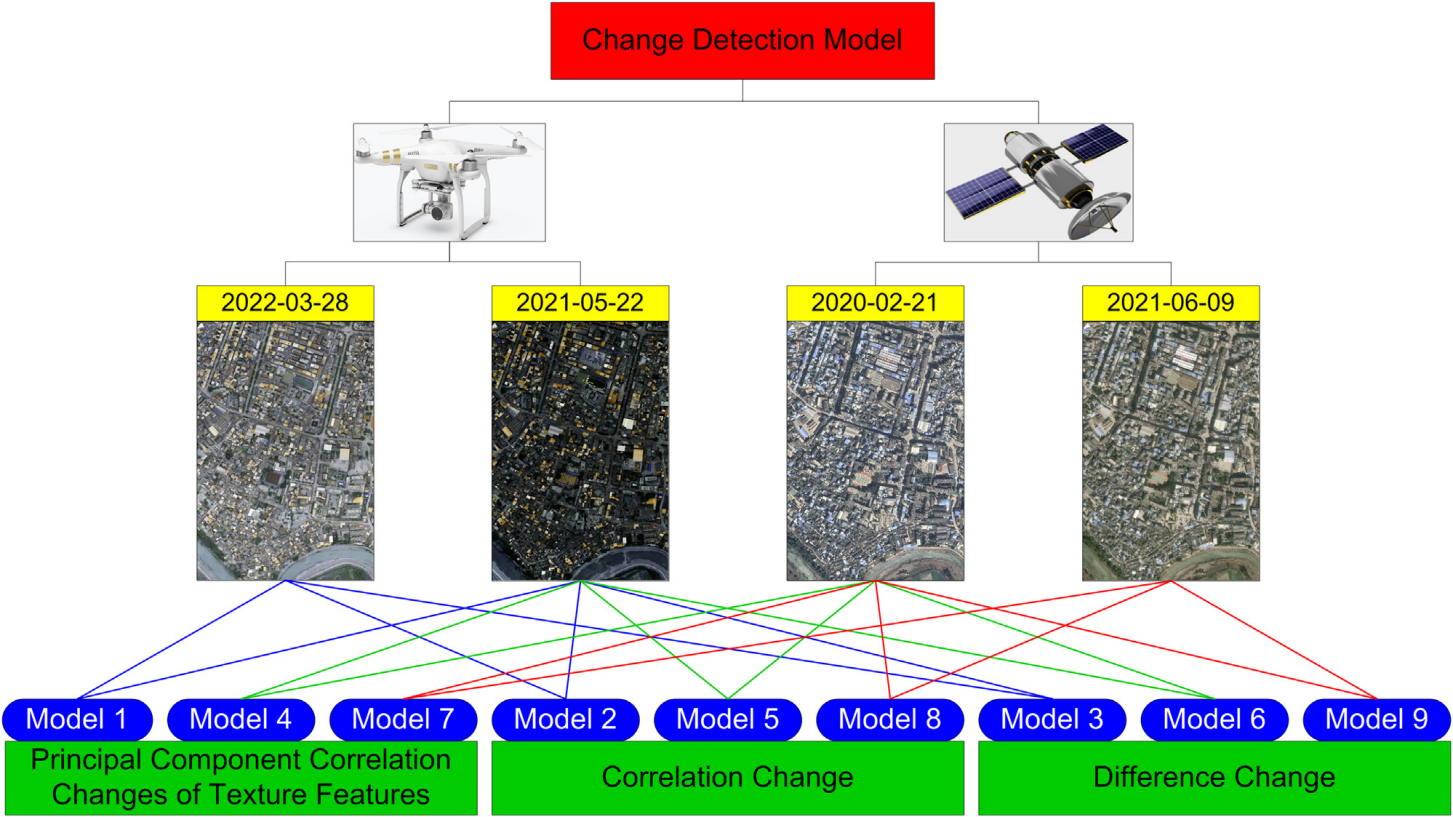
Image change detection model Image change detection was conducted for satellite images on February 21, 2020 (before the earthquake), UAV images on May 22, 2021 (after the earthquake), satellite images on June 9, 2021 (after the earthquake), and UAV images on March 28, 2022 (after repair of damaged buildings). This was based on the principal component correlation, correlation change, and difference change of the texture features. There were nine models in total, of which Model 4 was a verification model.

**Figure 2:**
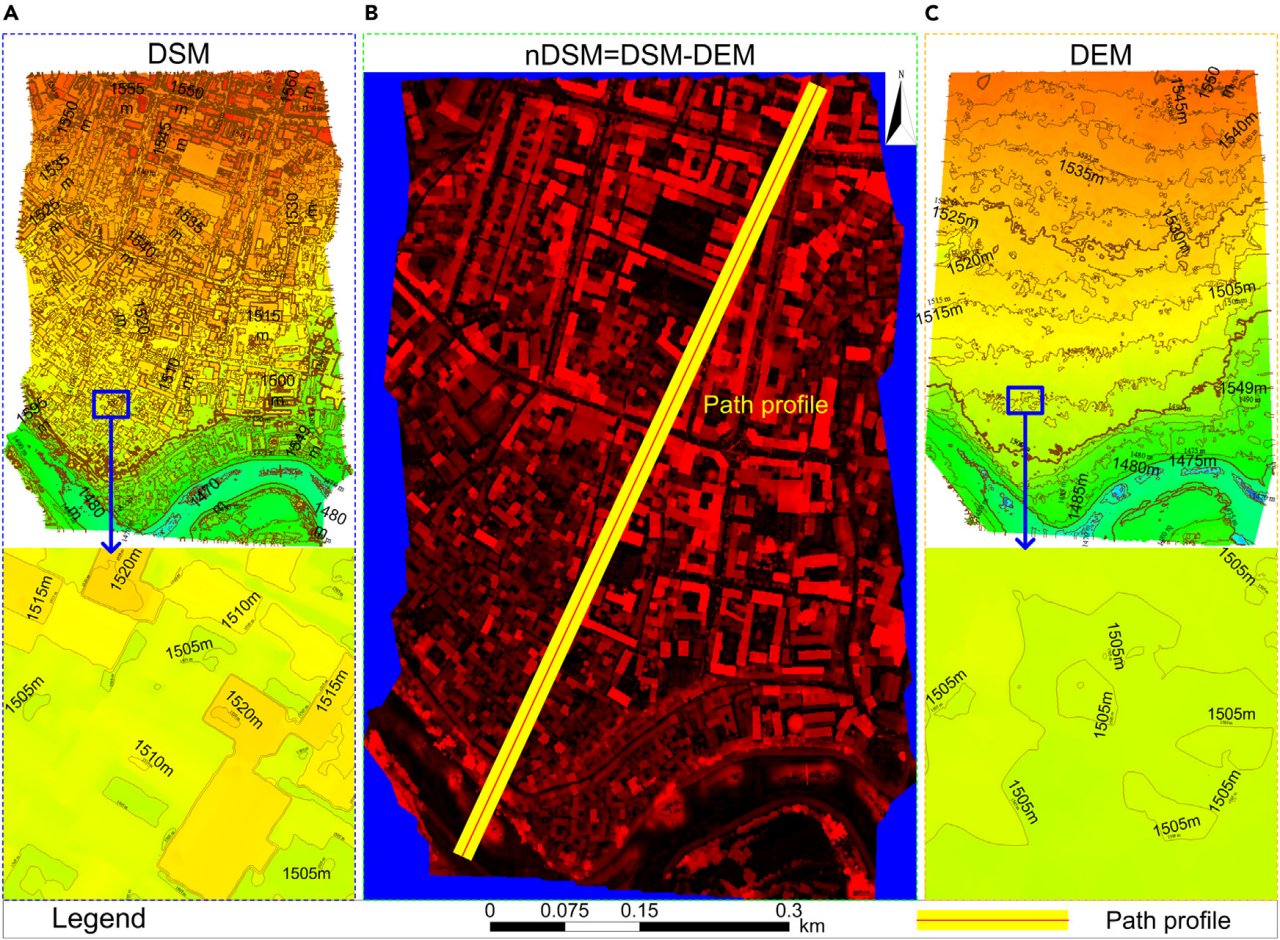
The solution results of nDSM (A) The DSM images obtained by UAV were converted into LiDAR LAS data to complete the gap repair; (B) the differential image nDSM is obtained by subtracting DEM from DSM; (C) using point cloud refinement to generate dense contour lines, select threshold to generate DEM with grid spacing of 0.3 m and expected terrain slope of 7.5° and extract DEM contour lines.

**Figure 3:**
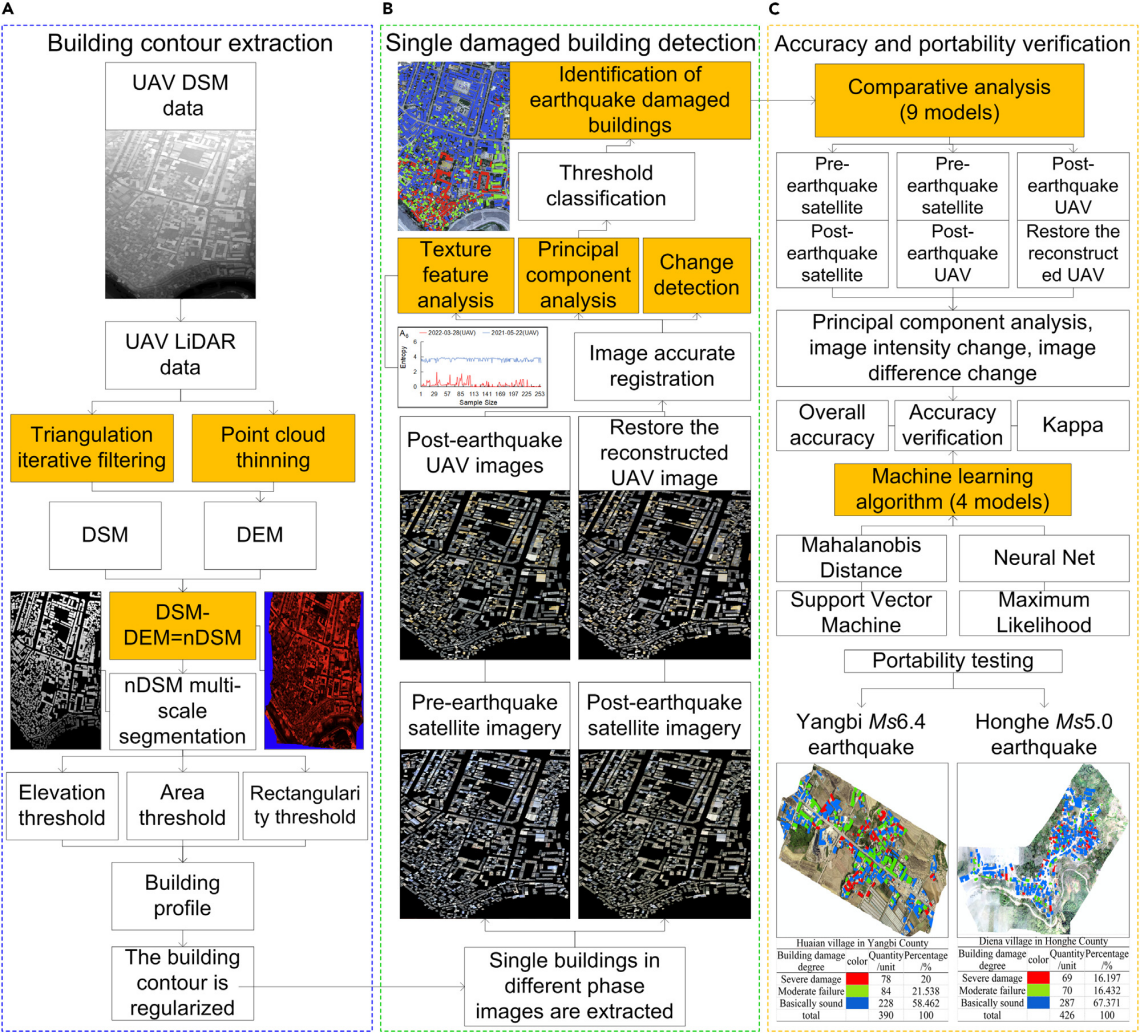
Model flow chart (A–C) (A) The nDSM image eliminates the influence of vegetation, terrain, and shadow and extracts the outline information of the single building; (B) building profile ROI data are used to generate single images of buildings before and after earthquakes from different data sources; (C) multi-feature combination and principal component changes are used to eliminate the information redundancy caused by multi-feature correlation and noise in images, so as to realize the identification, location, and classification of a single damaged building.

It is generally agreed that dual-temporal change detection comparing pre-earthquake with post-earthquake image data, the analysis of post-earthquake UAV DOM and nDSM images, and texture feature analysis play important roles in remote sensing (Figure 1). We combine the advantages of these three approaches to propose a single-building damage detection model based on multi-feature fusion to address the following problems: (1) insufficient samples in earthquake damage identification algorithms, (2) low method portability caused by the difference between the sample dataset and test data sensors, (3) inaccurate building boundaries in earthquake damage identification, (4) accurate location of single damaged buildings, and (5) elimination of the effect of changes in non-building information and the many texture feature parameters, which are difficult to optimize. Specifically, the proposed model aims to meet three objectives: (1) to extract the contour information of individual buildings by fully utilizing the post-earthquake UAV DOM and nDSM images and eliminating the influence of vegetation, terrain, and shadows through nDSM images (Figure 2); (2) to generate single pre-earthquake and post-earthquake images of buildings from different data sources using the building contour ROI data and to realize the fine registration of different building single images using the algorithm based on rational polynomial; and (3) to realize the identification, location, and classification of single damaged buildings by extracting texture feature parameters with good discrimination from different damaged buildings and calculating their correlations using principal component change to eliminate the information redundancy caused by multi-feature correlation and noise in the images. Fine image registration is an important pre-processing step before multi-temporal image change detection. In this experiment, fine registration was realized by cross-correlation, based on the fitting global transform geometric model and a fitted global transform model. The workflow is divided into three stages (Figure 3): (1) building contour extraction (A), (2) single damaged building detection (B), and (3) accuracy verification and portability test (C).

## Results

### Comparative analysis of detection results of damaged buildings through different models

**Figure 4:**
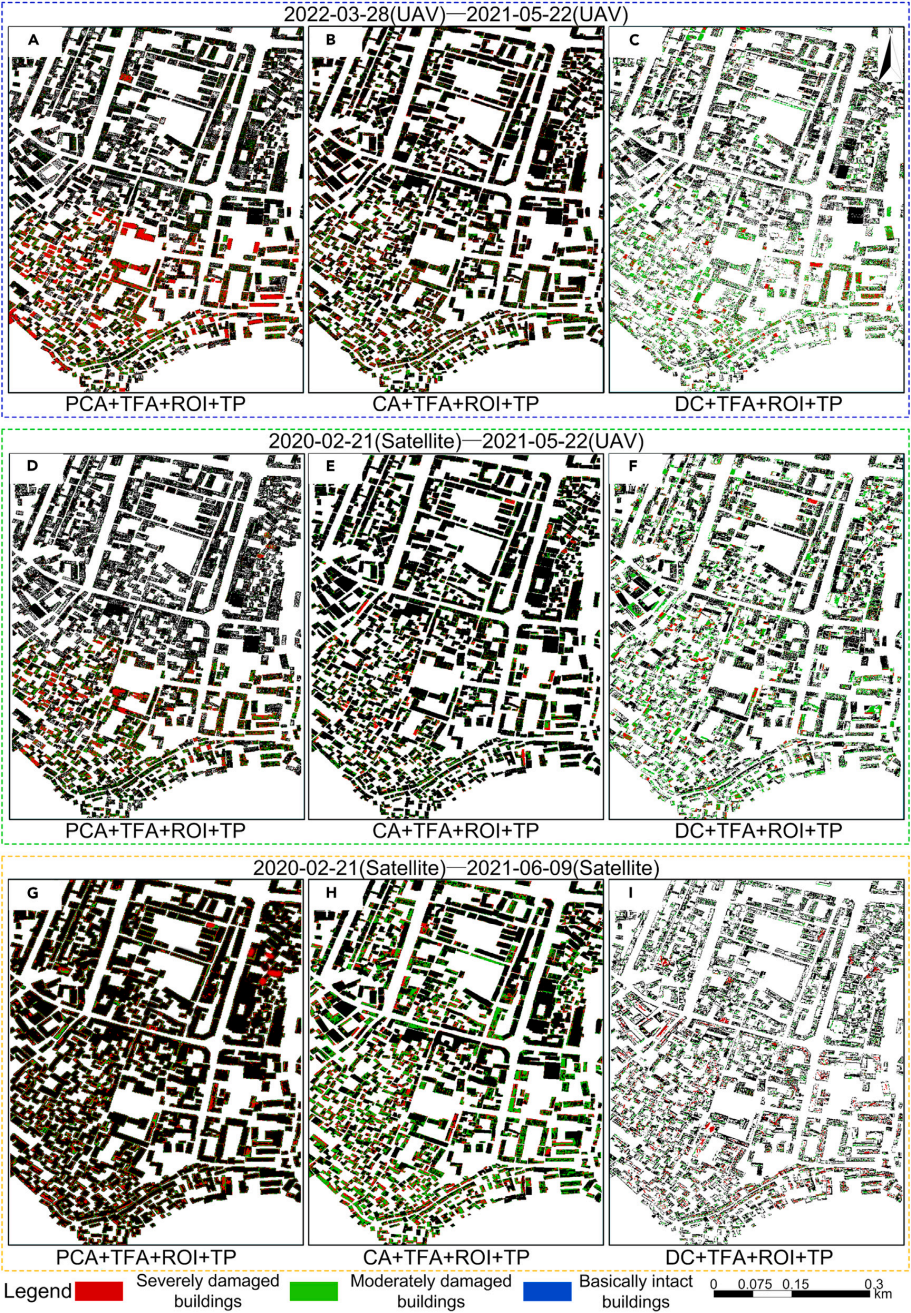
Extraction results of damaged buildings with different models (A) Model 1: building damage detection results based on the correlation of principal component changes in texture features of UAV images before and after the earthquake (PCA+TFA+ROI+TP); (B) Model 2: building damage detection results based on the correlation between texture features of UAV images before and after the earthquake (CA+TFA+ROI+TP); (C) Model 3: building damage detection results based on intensity images of texture features of UAV images before and after earthquake (DC+TFA+ROI+TP); (D) Model 4: building failure detection results based on the correlation of principal components of texture features in pre-earthquake satellite images and post-earthquake UAV images.(PCA+TFA+ROI+TP); (E) Model 5: building damage detection results based on the correlation between texture features of satellite images before earthquake and UAV images after earthquake (CA+TFA+ROI+TP); (F) Model 6: building damage detection results based on pre-earthquake satellite image and post-earthquake UAV image texture feature intensity image (DC+TFA+ROI+TP); (G) Model 7: building failure detection results based on the correlation of principal components of texture features in satellite images before and after earthquakes (PCA+TFA+ROI+TP); (H) Model 8: building failure detection results based on the correlation between texture features of satellite images before and after earthquakes (CA+TFA+ROI+TP); (I) Model 9: building damage detection results based on intensity images of texture features in satellite images before and after earthquakes (DC+TFA+ROI+TP).

**Figure 5:**
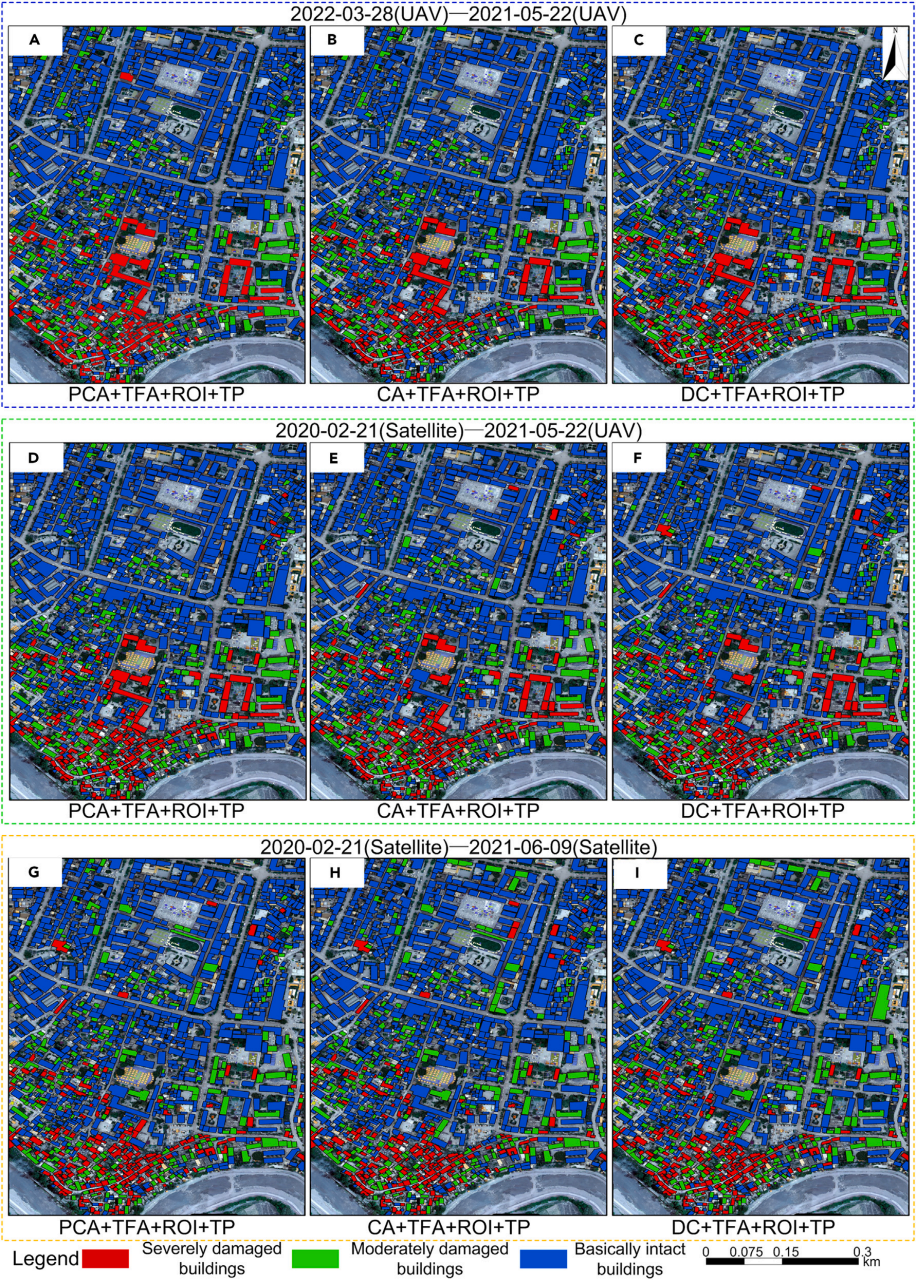
Distribution of damaged buildings in different models (A) Statistics and distribution results of damaged building detection using PCA+TFA+ROI+TP method (Model 1); (B) statistics and distribution results of damaged building detection using CA+TFA+ROI+TP method (Model 2); (C) statistics and distribution results of damaged building detection using DC+TFA+ROI+TP method (Model 3); (D) statistics and distribution results of damaged building detection using PCA+TFA+ROI+TP method.(Model 4); (E) statistics and distribution results of damaged building detection using CA+TFA+ROI+TP method (Model 5); (F) statistics and distribution results of damaged building detection using DC+TFA+ROI+TP method (Model 6); (G) statistics and distribution results of damaged building detection using PCA+TFA+ROI+TP method (Model 7); (H) statistics and distribution results of damaged building detection using CA+TFA+ROI+TP method (Model 8); (I) statistics and distribution results of damaged building detection using DC+TFA+ROI+TP method (Model 9).

Comparative analysis was conducted on the nine models (Figure 1) proposed in the paper through field research and visual interpretation of images. The principal component correlation change detection method based on texture features (Model 1, Model 4, Model 7) integrated multiple texture feature parameters from pre-earthquake and post-earthquake images. By calculating the correlation between two texture feature images, the earthquake-damaged buildings were classified according to the statistical correlation coefficient classification threshold. The correlation change detection method (Model 2, Model 5, Model 8) on two pre-earthquake and post-earthquake intensity images and then the earthquake-damaged buildings were classified according to the statistical correlation coefficient classification threshold. The image difference operation (Model 3, Model 6, Model 9) was performed on two pre-earthquake and post-earthquake images, and then the damaged building information was extracted by classifying the difference images according to the sample statistical threshold. The extraction results are shown in Figure 4. The classification of model earthquake damage shown in Table 1 was used as the standard. The proportion of damaged areas in each building contour, as shown in Figure 4, was calculated, and earthquake-damaged buildings were classified as intact, moderately damaged, and severely damaged. The earthquake damage distribution results for different models after merging are shown in Figure 5. The overall accuracy (OA) and κ coefficient for earthquake damage identification from 13 models were calculated. Table 2 shows the earthquake damage identification accuracies of the different models.

**Table 1:** Classification method of damage degree of single building

Damage grade	Color proportion			Result	Explain
	SD (Red)	MD (Green)	BI (Blue)		
SD	If (red＞10%)	Yes	Yes	SD	Roof collapse or large holes cause large changes in texture features.
MD	If (red≤10%)	If (green＞50%)	Yes	MD	The shingles drop resulting in slight changes in texture features.
BI	If (red≤10%)	If (green＞0% and green≤50%)	Yes	BI	Minor damage to shingles or no damage to roof.

BI, basically intact; MD, moderately damaged; SD, severely damaged.

**Table 2:** Evaluation result of single-building damage detection based on confusion matrix

Model				Prediction result					
Method	Number			BI (Blue)	MD (Green)	SD (Red)	Sum	OA (%)	Kappa
PCA+TFA+ROI+TP	1	Ground truth	BI	812	18	18	848	93.422	0.8702
			MD	13	199	9	221		
			SD	12	14	182	208		
			Sum	837	231	209	1277		
CA+TFA+ROI+TP	2		BI	811	17	20	848	92.717	0.856
			MD	13	197	11	221		
			SD	15	17	176	208		
			Sum	839	231	207	1277		
DC+TFA+ROI+TP	3		BI	806	19	23	848	92.09	0.8443
			MD	16	192	13	221		
			SD	12	18	178	208		
			Sum	834	229	214	1277		
PCA+TFA+ROI+TP	4		BI	790	25	33	848	89.271	0.79
			MD	21	182	18	221		
			SD	15	25	168	208		
			Sum	826	232	219	1277		
CA+TFA+ROI+TP	5		BI	785	27	36	848	88.801	0.7812
			MD	22	181	18	221		
			SD	17	23	168	208		
			Sum	824	231	222	1277		
DC+TFA+ROI+TP	6		BI	783	28	37	848	88.723	0.78
			MD	21	182	18	221		
			SD	18	22	168	208		
			Sum	822	232	223	1277		
PCA+TFA+ROI+TP	7		BI	771	40	37	848	85.904	0.7252
			MD	26	172	23	221		
			SD	24	30	154	208		
			Sum	821	242	214	1277		
CA+TFA+ROI+TP	8		BI	761	47	40	848	84.259	0.694
			MD	30	166	25	221		
			SD	26	33	149	208		
			Sum	817	246	214	1277		
DC+TFA+ROI+TP	9		BI	755	51	42	848	83.163	0.6732
			MD	33	161	27	221		
			SD	27	35	146	208		
			Sum	815	247	215	1277		

TFA, texture feature analysis; DC, difference change; CA, correlation analysis; TP, two phases; SP, single phase; BI, basically intact; MD, moderately damaged; SD, severely damaged.

**Figure 6:**
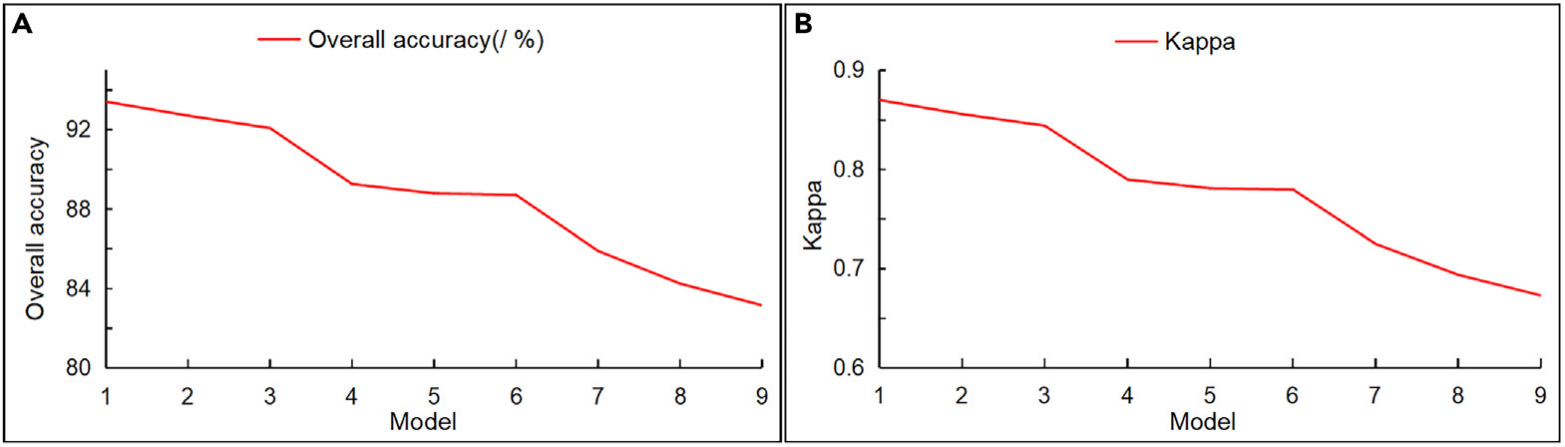
Trend curve (A) Overall accuracy trend curve. (B) Kappa accuracy trend curve.

The trend curves of overall identification accuracy and κ are shown in Figure 6. It can be concluded from Table 2 and Figure 5 that, firstly, the OA and κ coefficient of Models 1–9 show a decreasing trend. Secondly, the OA of Models 1–9 in identifying damaged buildings was above 80%, with good recognition effect, indicating that these models can effectively identify intact and damaged buildings. The OA and κ coefficient of different data sources were ranked from high to low as follows: post-earthquake and reconstruction UAV images (Models 1, 2, 3) > pre-earthquake satellite images and post-earthquake UAV images (Models 4, 5, 6) > pre-earthquake and post-earthquake satellite images (Models 7, 8, 9). Clearly, image resolution plays an important role in the detection process.

The OA and κ coefficient of the same data sources were ranked from high to low as follows: PCA+TFA+ROI+TP (Models 1, 4, 7) > CA+TFA+ROI+TP (Models 2, 5, 8) > DC+TFA+ROI+TP (Models 3, 6, 9). The reason is that texture features based on statistical analysis can suppress the impact of speckle noise. Therefore, principal component correlation resulted in fewer misclassification phenomena, whereas speckle noise affected the other two methods, reducing their accuracy.

## Discussion

### Effectiveness of damage assessment results

**Figure 7:**
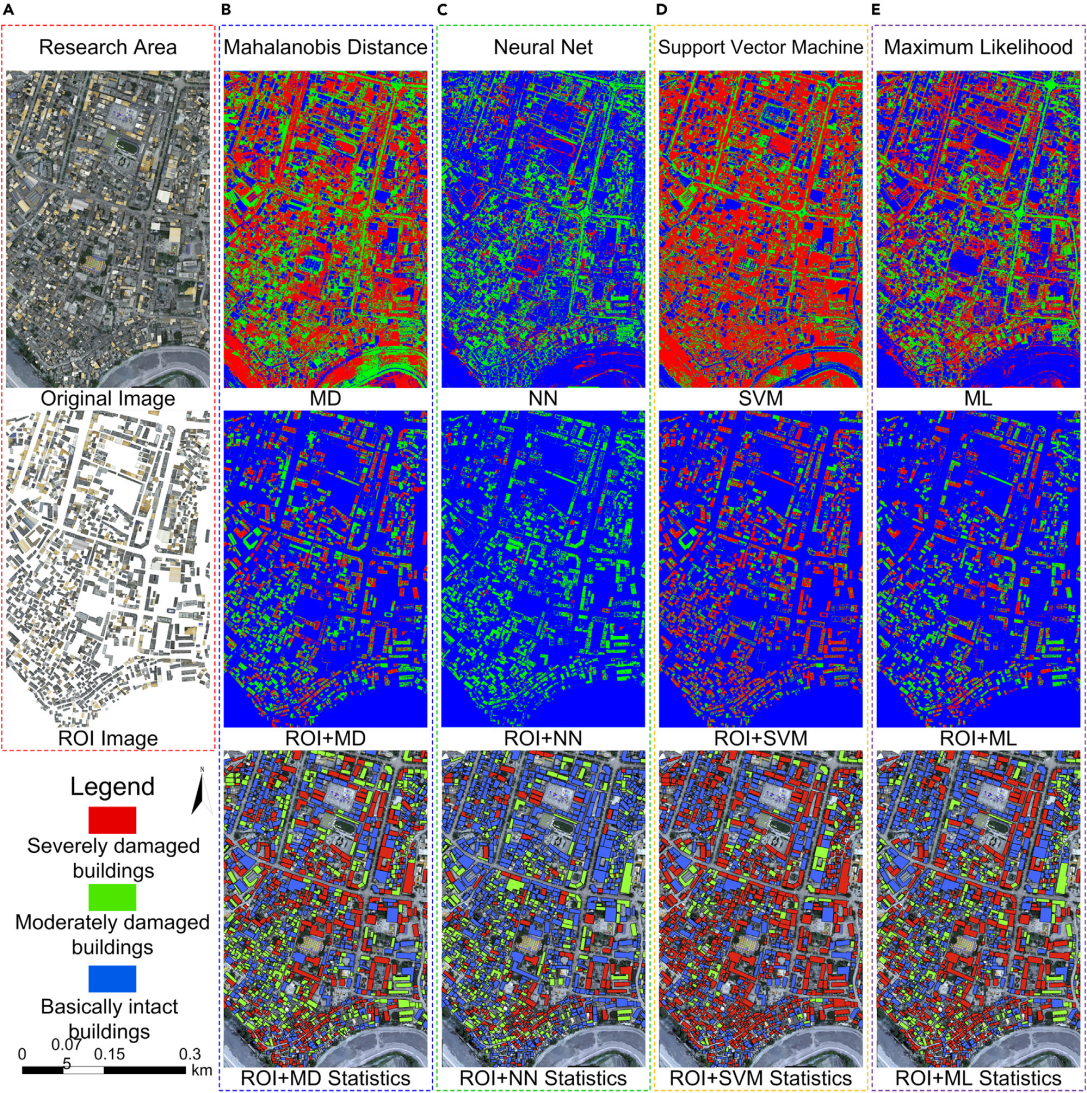
Damage detection results of different models of buildings (A) Study regional DOM image and regional ROI+DOM image; (B) building earthquake damage identification results based on MD, ROI+MD algorithm, and ROI+MD statistical results; (C) building earthquake damage identification results based on NN, ROI+NN algorithm, ROI+NN statistical results; (D) identification results of building earthquake damage based on SVM, ROI+SVM algorithm, and statistical results of ROI+SVM; (E) building earthquake damage identification results based on ML, ROI+ML algorithm, and ROI+ML statistical results.

To assess the effectiveness of PCA + TFA + ROI + TP (Model 4), maximum likelihood classification (ML), neural net classification (NN), Mahalanobis distance classification (MD), and support vector machine classification (SVM) machine learning models were selected for comparison. Overall, 100 building samples were selected and marked for accurate evaluation. Table 3 shows the detection results based on pixels. According to Figure 7, if ML, NN, MD, and SVM models were directly used, there was considerable non-building information and unclear building contours in the detection results, failing to locate and detect damaged buildings. When ROIs were used, there were considerable improvements in the building contours and detection effects. The identification, location, and classification of damaged buildings were realized when using ROIs (Table 4), but such a method led to more misclassified pixels in classification results compared with the dual-phase change detection method, confirming the effectiveness of Model 4.

**Table 3:** Pixel statistic results of building damage detection based on MD, NN, SVM, and ML methods

Method	Prediction result			Scale (%)		
	BI (Blue)	MD (Green)	SD (Red)	BI (Blue)	MD (Green)	SD (Red)
ROI+MD+SP	181602009	33903920	28389445	74.45	13.9	11.64
MD + SP	59034625	74860057	110000692	24.2	30.69	45.1
ROI+NN+SP	1974496	426373	37179	80.99	17.49	1.52
NN + SP	1913292	763273	363140	62.94	25.11	11.95
ROI+SVM+SP	181882882	23553654	38458838	74.57	9.65	15.77
SVM+SP	59633597	54222494	130039283	24.45	22.23	53.22
ROI+ML+SP	191693361	28078481	24123532	78.59	11.51	9.89
ML + SP	111102376	53715938	79077060	45.55	22.02	32.42

ROI, region of interest; SP, single phase; ML, maximum likelihood classification; NN, neural net classification; MD, Mahalanobis distance classification; SVM, support vector machine classification; BI, basically intact; MD, moderately damaged; SD, severely damaged.

**Table 4:** Statistical results of building damage detection based on MD, NN, SVM, and ML methods

Model				Prediction result					
Method	Number			BI (Blue)	MD (Green)	SD (Red)	Sum	OA (%)	Kappa
ROI+ML+SP Statistics	10	Ground truth	BI	483	137	228	848	57.17	0.316
			MD	36	102	83	221		
			SD	12	51	145	208		
			Sum	531	290	456	1277		
ROI+NN+SP Statistics	11		BI	613	146	89	848	61.24	0.276
			MD	82	95	44	221		
			SD	67	67	74	208		
			Sum	762	308	207	1277		
ROI+MD+SP Statistics	12		BI	408	218	222	848	48.86	0.218
			MD	33	96	92	221		
			SD	18	70	120	208		
			Sum	459	384	434	1277		
ROI+SVM+SP Statistics	13		BI	412	99	337	848	46.81	0.183
			MD	37	11	173	221		
			SD	22	8	172	202		
			Sum	471	118	682	1271		

ROI, region of interest; SP, single phase; ML, maximum likelihood classification; NN, neural net classification; MD, Mahalanobis distance classification; SVM, support vector machine classification; BI, basically intact; MD, moderately damaged; SD, severely damaged.

### Reliability of damage assessment results

**Figure 8:**
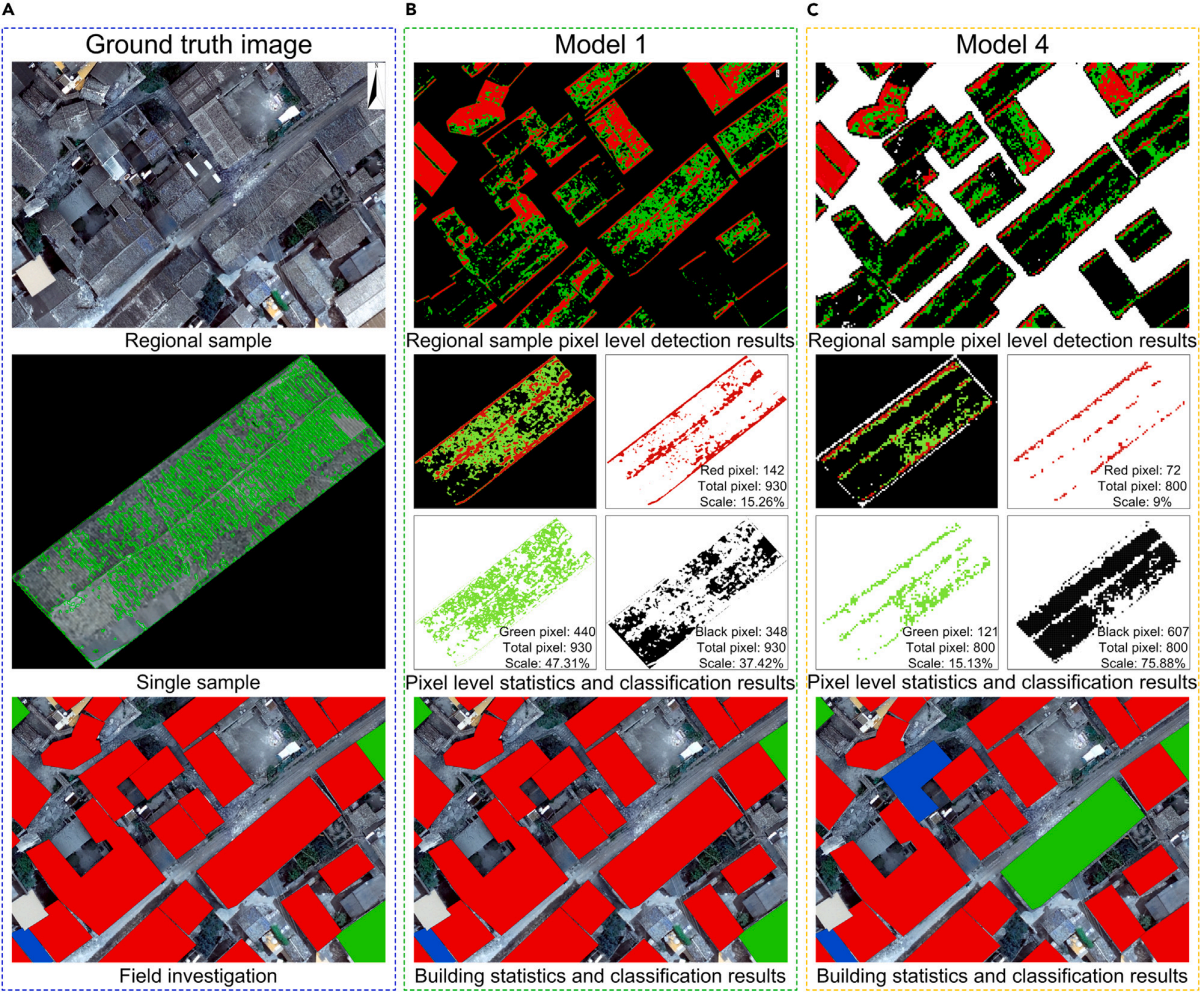
Model 1 and Model 4 detect damaged buildings and analyze the results (A) Results of field investigation and visual interpretation of images; (B) Model 1 used PCA+TFA+ROI+TP to detect the earthquake damage of UAV images before and after the earthquake (Red pixel: 142, Scale: 15.26%; Green pixel: 440, Scale: 47.31%; Black pixel: 348, Scale: 37.42%); (C) Model 4 uses PCA+TFA+ROI+TP seismic damage detection results of pre-earthquake satellite image and post-earthquake UAV image (Red pixel: 72, Scale: 9%; Green pixel: 121, Scale:15.13%; Black pixel: 607, Scale: 75.88%).

The test results showed that Model 1 had the best change detection effect for damaged buildings. Specifically, as shown in Figure 8, Model 1 performed better at recognizing roof cracks and shuttle tiles. This model relies, however, on pre-earthquake UAV images, which can be difficult to obtain because they are not routinely collected. The good performance of Model 4 (inferior only to Model 1, but much higher than that of the two pre-earthquake and post-earthquake satellite images) illustrates how introducing post-earthquake UAV nDSM images eliminated the impact of non-building changes, greatly improving the accuracy of earthquake damage identification. Although Model 4 was able to detect most buildings with damaged roofs, the damage detection pixels are sparser than those from Model 1. There are two main reasons. (1) Differences in image quality: The quality of satellite images is different from that of UAV images. Satellite images may have problems, such as blur and noise, owing to limitations by atmospheric conditions, cloud covering, and other factors, whereas UAV images have a higher resolution and clarity owing to the close range. Compared with the pre-earthquake and post-earthquake images obtained by the same sensor on the UAV (Model 1), some errors in the change detection of images occurred with different quality in Model 4. (2) Image registration: Due to different data sources, pre-earthquake satellite images and post-earthquake UAV images should be recorded for effective comparison. However, this may be difficult because of terrain complexity and image distortion, and some recording errors may be introduced.

### Effectiveness of nDSM in injury assessment

**Figure 9:**
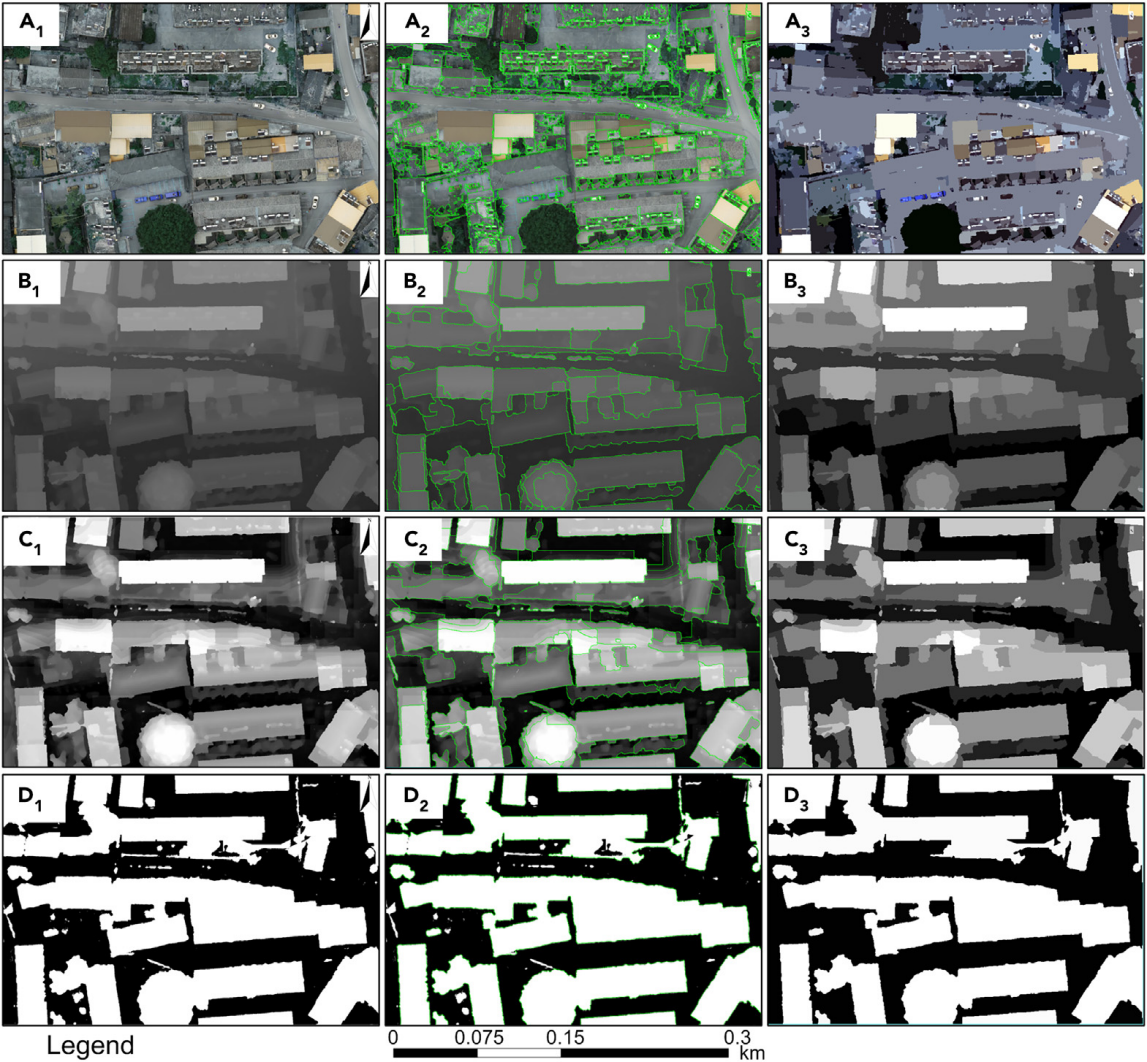
Results of building contour extraction from different images (A_1-3_) Building contour extraction results based on DOM image; (B_1-3_) building contour extraction results based on DSM images; (C_1-3_) building contour extraction results based on nDSM image; (D_1-3_) building contour extraction results based on nDSM binary image.

As shown in Figure 9, DOM (a_1_), DSM (b_1_), nDSM (c_1_) and nDSM binary images (d_1_) of the same area were segmented at multiple scales to verify that the nDSM binary image performed well at extracting building contours. The multi-scale segmentations of DOM images (Figure 9A_3_) exhibit poor distinguishability of some ground and road areas, as their image feature values are similar to those of buildings. In addition, the small image segmentation led to over-classification, as it was affected by elements such as solar water heaters on roofs.

Such overclassification of non-building objects was avoided in the multi-scale segmentations of DSM images (Figure 9B_3_). However, some buildings are highly consistent with the undulating terrain, making it impossible to distinguish the ground, roads and buildings over large scales.

Terrain fluctuations are addressed well by the multi-scale segmentations of nDSM images (Figure 9C_3_), but some building contours are over-classified due to the fusion of contour lines with some building roofs in DEM, affecting the extraction of building contours.

Multi-scale segmentation of nDSM binary images (Figure 9D_3_) not only eliminated the over-classification of ground objects, but also eliminated the fusion of contour lines and some building roofs through threshold segmentation. In addition, small scattered fragments were filtered out. This method demonstrated good performance in extracting building contours because it decisively extracts individual buildings from different image data sources.

### Effectiveness of texture features in damage assessment

The changes in buildings in the images are complex and diverse, affecting the correlation of their features. However, building damage can be more effectively reflected by texture features, and the feature changes of images with different data sources and captured at different times are more stable. Therefore, the correlation change detection of texture features was adopted in this research. Feature factors can exert either positive or negative effects on image change detection due to the large of number of factors involved in describing texture features. Therefore, optimal feature fusion and PCA methods was adopted to select the optimal feature subset, avoiding false detection caused by information redundancy, and effectively improving the accuracy of earthquake damage detection of buildings.

**Figure 10:**
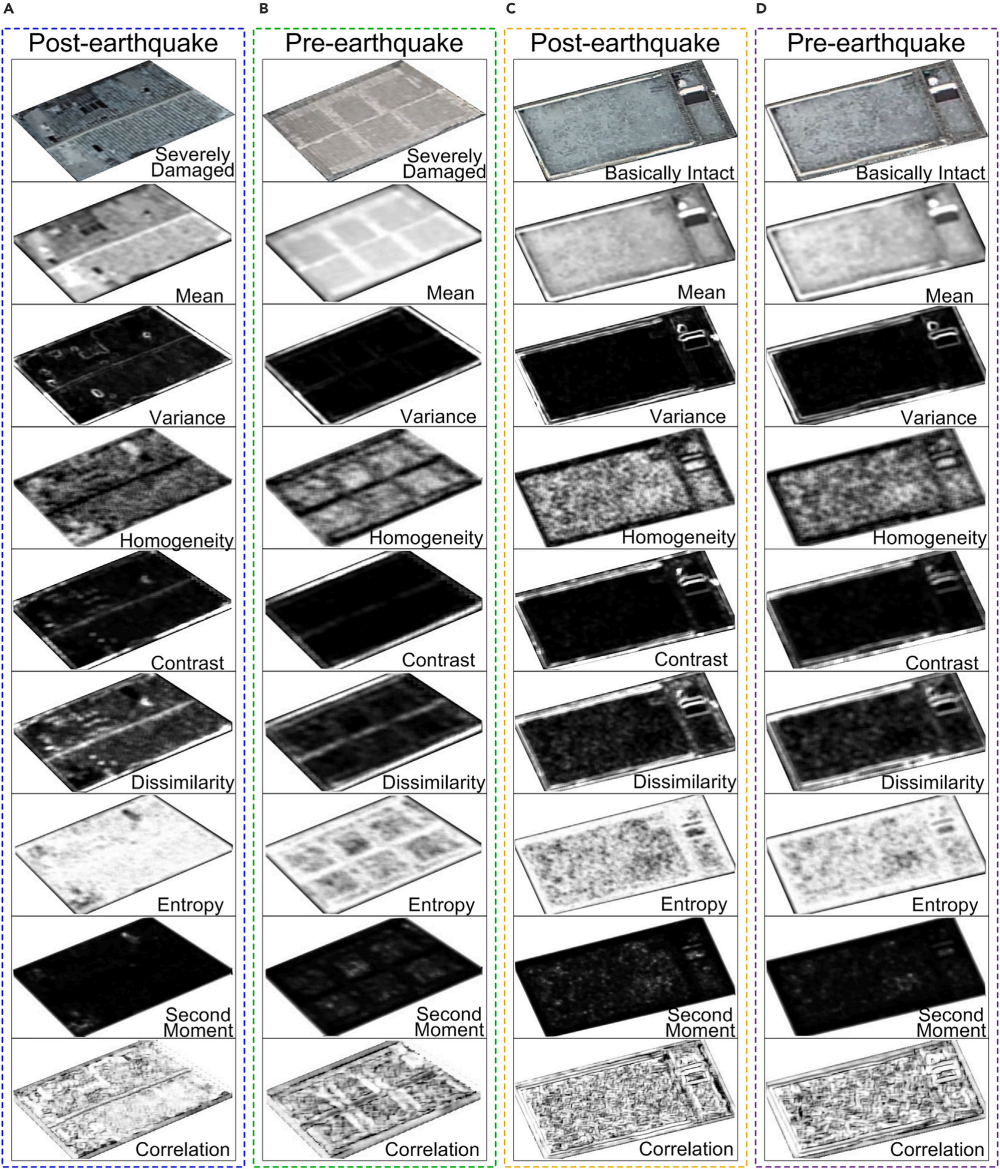
The feature maps of the damaged and basically intact buildings were compared (A) Samples of severely damaged buildings after the earthquake and eight texture feature maps; (B) samples of severely damaged buildings and eight texture maps before the earthquake; (C) samples of heavily damaged buildings after the earthquake and eight texture feature maps; (D) samples of severely damaged buildings before the earthquake and eight texture feature maps.

**Figure 11:**
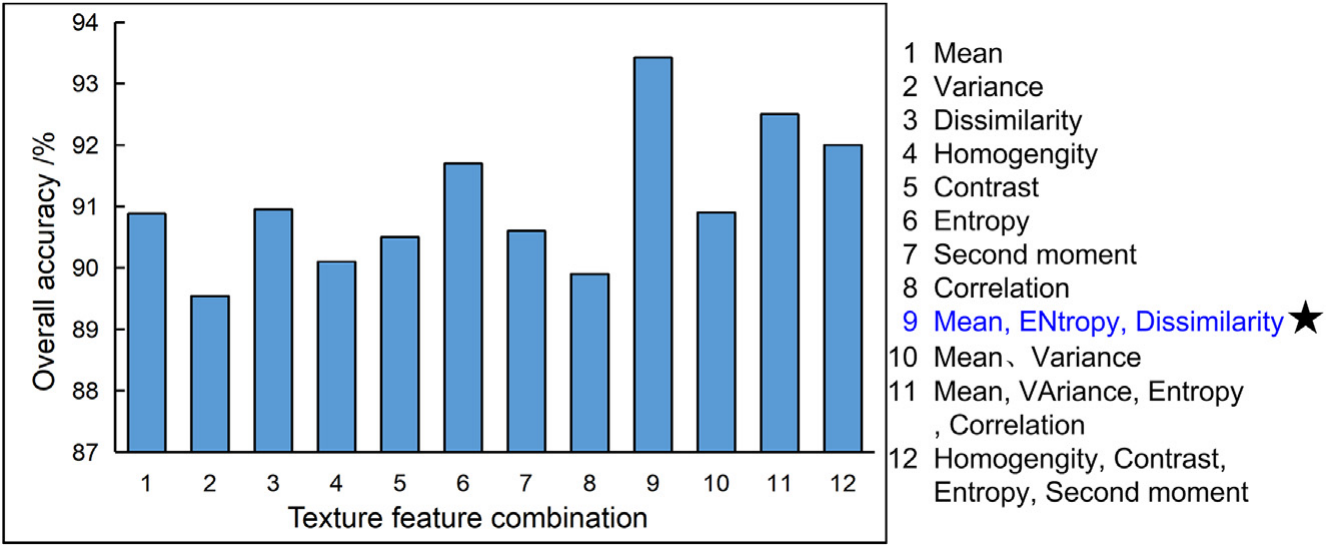
Comparison of training accuracy based on different texture feature combinations There are significant differences in the overall accuracy of different feature vector combinations. Therefore, the selected features in this research were mean, entropy, and dissimilarity.

The feature map of the damaged and basically intact buildings were compared (Figure 10). On the basis of model 1, different texture feature combinations are added, and the classification method in Table 1 is used for experiments. As can be seen in Figure 11, there are significant differences in the overall accuracy of different feature vector combinations. Therefore, the selected features in this research were mean, entropy, and dissimilarity.

### Model portability testing

**Figure 12:**
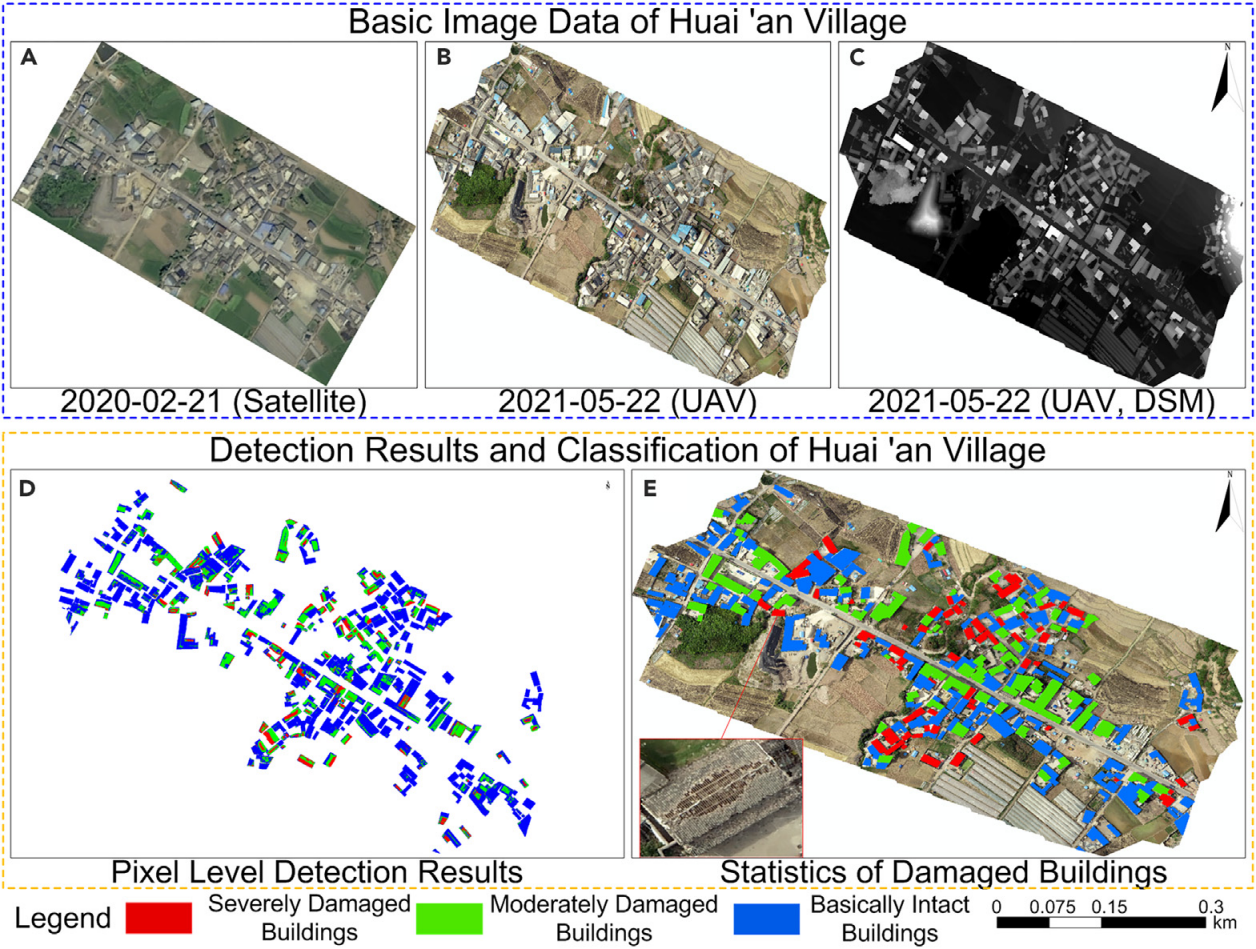
Earthquake damage detection results of buildings in Huai'an Village (A) Satellite DOM image on February 21,2020; (B) UAV DOM image on May 22, 2021; (C) UAV DSM image on May 22, 2021; (D) Detection results and classification of Huai'an village based on pixel level; (E) building damage statistics and distribution results.

**Figure 13:**
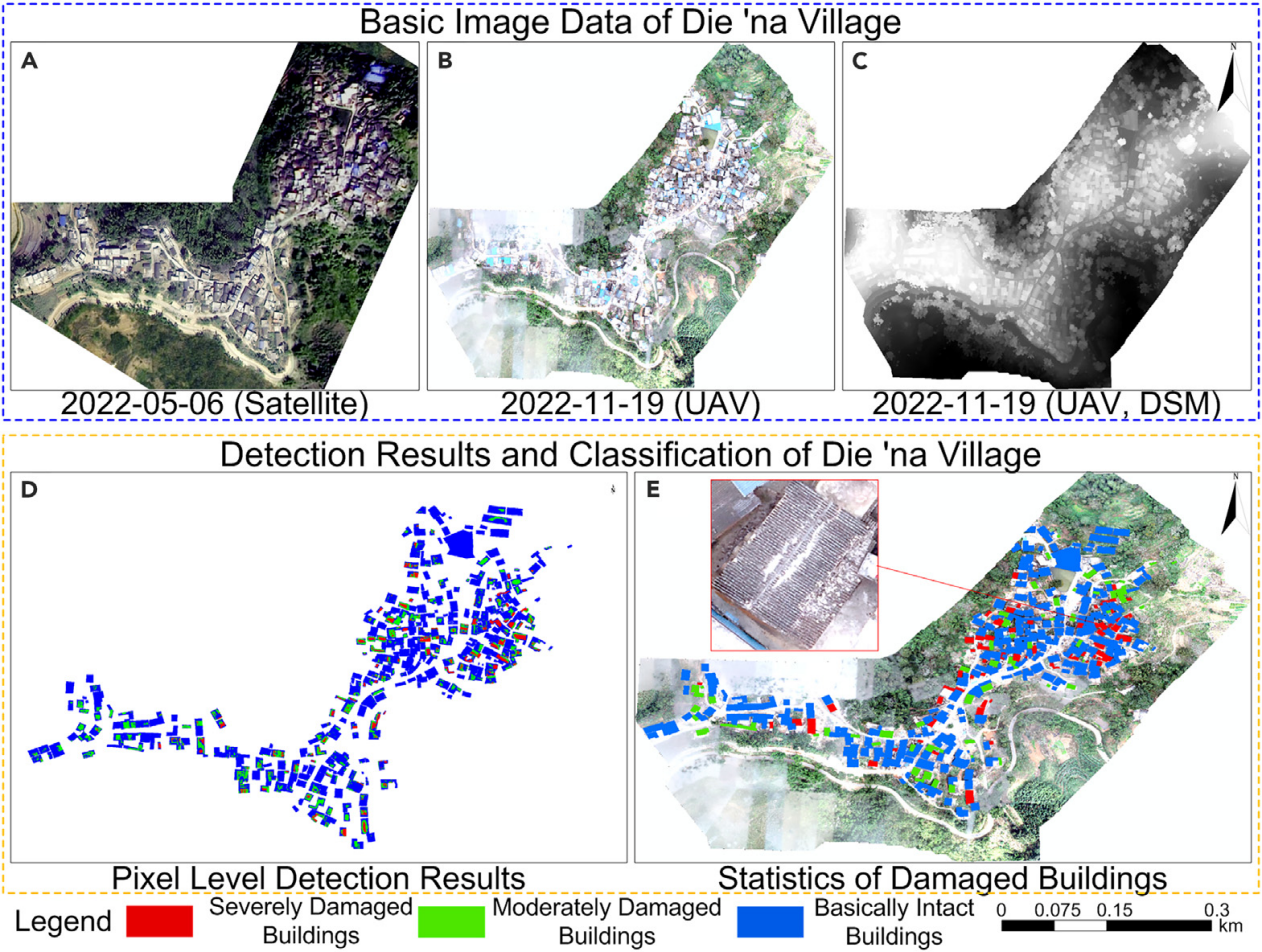
Earthquake damage detection results of buildings in Die'na Village (A) Satellite DOM image on May 6, 2022; (B) UAV DOM image on November 19, 2022; (C) UAV DSM image on May 22, 2021; (D) detection results and classification of Die'na village based on pixel level; (E) building damage statistics and distribution results.

The model proposed in this study was applied to the Yangbi *M*_*S*_6.4 earthquake that occurred on May 21, 2021, in Huai'an Village and the Honghe *M*_*S*_5.0 earthquake on November 19, 2022, in Die'na Village, China, and the earthquake damage results were verified. Figures 12 and 13 show the post-earthquake damage detection results for single buildings based on multi-feature fusion in Huai'an Village and Die'na Village. According to the confusion matrix shown in Table 5, the average accuracy for building earthquake damage identification was 89.066%, and the average κ coefficient was 0.7995, suggesting that the model has good recognition accuracy.

**Table 5:** Detection results and accuracy of damaged buildings in Huai'an and Die'na Village

				Prediction result					
Model	Region			BI (Blue)	MD (Green)	SD (Red)	Sum	OA (%)	Kappa
4	Huai'an Village	Ground truth	BI	208	13	7	228	88.461	0.801
			MD	5	70	9	84		
			SD	4	7	67	78		
			Sum	217	90	83	390		
	Die'na Village		BI	263	13	11	287	89.671	0.797
			MD	7	58	5	70		
			SD	3	5	61	69		
			Sum	273	76	77	426		

BI, basically intact; MD, moderately damaged; SD, severely damaged.

### Timeliness estimation

To evaluate the efficiency of our workflow, we performed a timeliness analysis in three sites (Yangbi County, Huai'an Village, and Die'na Village). The first stage of the analysis takes approximately 3 h (UAV image acquisition + data preprocessing), the second stage takes approximately 1 h (detection of single damaged buildings), and the third stage takes approximately 30 min (identification, positioning, and classification of the damage to single buildings in the earthquake area). To detect the damage and obtain the final assessment results, it takes approximately 4.5 h. However, the processing time can only be estimated under certain conditions, and the efficiency of evaluation may be affected by the characteristics of different seismic regions and experimental equipment.

### Conclusions

In this paper, a single-building damage detection model based on multi-feature fusion was proposed. Using the Yangbi *M*_*S*_6.4 earthquake and Honghe *M*_*S*_5.0 earthquake as examples, we tested the model with different data sources using nine change detection models, including PCA + TFA + ROI + TP, CA + TFA + ROI + TP, and DC + TFA + ROI + TP. Additionally, the model was compared with four machine learning algorithms (ROI + ML + SP, ROI + NN + SP, ROI + MD + SP, and ROI + SVM + SP). The accuracy and effectiveness of building damage detection based on PCA + TFA + ROI + TP (Model 4) were verified based on pre-earthquake satellite images and post-earthquake UAV images. The proposed model has the following four characteristics: (1) nDSM is used to extract building contours, improving the accuracy of this extraction. (2) ROIs are employed to generate single-building images from different data sources, eliminating the influence of non-building information, such as vegetation, terrain, and shadows, in the change detection to record accurate images. (3) Multi-feature fusion and principal component change detection are utilized, repressing information redundancy caused by multi-feature correlation and reducing noise in the images. (4) In contrast to pixel-based earthquake damage information presentation, the single-building-based presentation can separate earthquake damage types and display them as independent entities, enabling clearer observation and analysis of the damage to regional buildings.

In summary, a single-building damage detection model based on multi-feature fusion proposed in this paper can rapidly and accurately identify, locate, and classify damage to single buildings in earthquake areas. In the case of severe earthquake damage and urgent disaster relief tasks, the research results presented in this paper can provide high-precision data for post-earthquake loss assessment in addition to providing a decision-making basis for rapidly determining the locations of potential buried survivors and deploying rescue forces after the earthquake.

### Limitations of the study

Further research is needed to expand the models to cope with the diversity of data from multiple platforms and sensors after an earthquake. Comprehensive extraction methods should be considered for buildings described by different data sources and with varying degrees of damage. In this study, only a small number of moderately and severely damaged buildings were sampled in Yangbi County, Huai'an Village, and Die'na Village. In the future, more sample data should be obtained to improve the damage detection model of a single building based on multi-feature fusion and improve the generalization ability of the test model. An automated single-building information extraction, and automated selection of the optimal combination of multi-texture features of buildings, will be developed in the near future with the continuous advancement of deep learning, which will further improve the accuracy and efficiency of detecting damaged buildings after earthquake.

## STAR★Methods

### Key resources table

**Table:** 

REAGENT or RESOURCE	SOURCE	IDENTIFIER
**Deposited data**		

Binary map of buildings in Yangbi County	This manuscript	https://doi.org/10.6084/m9.figshare.24418732.v1
Outlines of buildings in Yangbi county	This manuscript	https://doi.org/10.6084/m9.figshare.24418777.v1
Satellite DOM image; 2020-02-21	This manuscript	https://doi.org/10.6084/m9.figshare.24418321.v1
Satellite DOM image; 2021-06-09	This manuscript	https://doi.org/10.6084/m9.figshare.24418217.v1
UAV DOM image; 2021-05-22	This manuscript	https://doi.org/10.6084/m9.figshare.24418144.v1
UAV DOM image; 2022-03-28	This manuscript	https://doi.org/10.6084/m9.figshare.24420097.v1
UAV DSM image; 2021-05-22	This manuscript	https://doi.org/10.6084/m9.figshare.24420100.v1
UAV nDSM image; 2021-05-22	This manuscript	https://doi.org/10.6084/m9.figshare.24420103.v1
Satellite image	Google Maps	http://www.wxno.com/

**Software and algorithms**		

Global_mapper 23.0	Global Mapper	http://www.globalmapper.com
ArcMap 10.2	Esri	http://www.esri.com
Omap	Ovital	http://www.ovital.com
eCognition	eCognition	https://www.ecognition.com
ENVI 5.3	Envi-met	https://www.envi-met.com/
DJI Terra	DJI	https://www.dji.com/cn

### Resource availability

#### Lead contact

Further information and requests for resources and materials should be directed to and will be fulfilled by the lead contact, Haoguo Du (1364125834@qq.com).

#### Materials availability

This study did not generate new unique reagents.

#### Data and code availability

As of the date of publication, drone images and satellite image data are publicly available. The data is stored in figshare and the doi is listed in the key resources table. If you have any reasonable request, please contact Hao Guo Du (e-mail:1364125834@qq.com) to obtain.

All data analysis is based on publicly available software packages (see methodology details), and the software installation website is listed in the key resources table. Any additional information necessary to reanalyze the data reported in this article can be obtained from the primary contact, Haoguo Du (e-mail:1364125834@qq.com).

### Experimental model and subject details

This study does not use experimental methods typical in the life sciences.

### Method details

#### Research area and data

The *M*_*S*_6.4 earthquake occurred at 21:48 on May 21, 2021 in Yangbi County (99.87° E, 25.67° N), Dali Prefecture, Yunnan Province, China, with a focal depth of 8 km, causing 3 deaths and 34 injuries. Yunnan sits on the eastern edge of the collision zone between the Indian Plate and the Eurasian Plate and experiences intense tectonic movement: its seismic activity is famous for high frequency, large magnitude, and wide distribution, raising particular interest from both domestic and foreign researchers. Yangbi County is located in the VIII intensity area. Its houses were damaged to varying degrees: a few walls of frame-structure houses cracked, and civil structure houses had, in general, partially collapsed.^44^^,^^45^ Because of its high urban population density and complex and dense housing structure, it is important to obtain accurate information on damaged buildings promptly after an earthquake. Figure S1 displays the UAV and satellite image data obtained in this study. The obtained image information is shown in Table S1. Figure S1A shows the result of 12,840 UAV images stitched using DJI Terra. Figure S1B shows the DSM images derived from corresponding UAV DOM images, which are based on the DSM dense point cloud obtained from the sparse point cloud using the multi-view stereo reconstruction method.

Figure 1 shows the image change detection model for which image change detection was conducted for satellite images on February 21, 2020 (before the earthquake), UAV images on May 22, 2021 (after the earthquake), satellite images on June 9, 2021 (after the earthquake), and UAV images on March 28, 2022 (after repair of damaged buildings). This was based on the principal component correlation, correlation change, and difference change of the texture features. There were nine models in total, of which Model 4 was a verification model. It is difficult to obtain UAV images before earthquakes owing to the uncertainty of earthquakes. Therefore, in this study, pre-earthquake UAV images were simulated based on UAV images on March 28, 2022 (after repair of damaged buildings) to complete the change detection, and the results of change detection of pre- and post-earthquake UAV images were tested. The parameters for the image acquisition device are shown in Table S2.

#### Extraction of nDSM building contour

The DSM contains information on buildings, trees, vegetation, water bodies, roads, open spaces, and other elements in addition to that in the DEM. The building contours on DSM images are clear, but the ground and ground objects on the images cannot be effectively distinguished by setting an elevation threshold, which will increase the interference to building contour extraction algorithms and lead to errors. For this reason, nDSM, the difference between UAV DSM and DEM, was used to extract building contour information in this study. This can effectively eliminate the factors making buildings in different regions show the same elevation to the ground due to terrain fluctuation and step, thus placing all ground objects on the same horizontal plane (Figure S2).

Firstly, the DSM images of the earthquake area obtained by the UAV were converted into LiDAR LAS data in this paper, and the extremely low and high points in the LiDAR LAS data were removed to complete gap reparation. Secondly, the triangular network iterative filtering method was used to distinguish ground points from non-ground points. Then, point cloud thinning was used to generate dense contour lines, and a threshold was selected to generate a DEM with a grid spacing of 0.3 m and an expected terrain slope of 7.5°, and DEM contour lines were extracted. The elevation threshold h was set to 3 m because the buildings are generally higher than 3 m. Finally, the difference image nDSM was obtained by subtracting DEM from DSM (Figure 2). Figure S3 shows the results of DSM, DEM and nDSM path profiles.

The process of building contour extraction based on nDSM is divided into three steps: nDSM multi-scale segmentation, construction of nDSM feature space, and small area removal method and the morphological closure operation.

#### nDSM multi-scale segmentation

Image segmentation, an early step of image-based analysis, was adopted to generate consistent objects. Only through image segmentation can image objects be obtained, forming the basic unit of object-level classification.^46^ In this study, ENVI5.3 was employed for sample classification in the study area using K-nearest neighbor, followed by multi-scale segmentation and merging, to acquire the optimal segmentation and merging scale for images in the study area. Figure S4 shows the results of multi-scale segmentation and merging of samples. When the segmentation and merging scale was less than 50, the samples were over-segmented (yellow box), affecting subsequent data processing. Hence, the segmentation and merging scales adopted were 90 and 90, 80 and 80, and 60 and 60, respectively, which are discussed below.

#### Construction of nDSM feature space

The spectral average grayscale value（Avgband_X）, rectangularity (Rect_fit) features and area (Area) were used to classify nDSM images in this paper. The image’s spectral average grayscale value is^33^

(Equation 1) $${\bar{A}}_{i}=\frac{1}{n}\sum_{i=1}^{n}A_{x\left(i\right)}$$

where *x* is the band number, *x*_(*i*)_ is the pixel value of the *i*^th^ pixel in the *x*^th^ band, with a range of (0, 255), and *n* is the number of pixels in the image.

The image’s rectangularity is

The rectangularity of the nth segmented pixel is

(Equation 2) $$Rect\left(n\right)=\sum_{i=1}^{n}\frac{S\left(i\right)}{R_{\max}\left(i\right)\cdot R_{\min}\left(i\right)}$$

where the value of the rectangle is 1 and the value of the non-rectangle is less than 1. *R*_min_(*i*) and *R*_max_(*i*) are the minimum and maximum width, respectively, of the *i*^th^ segmented pixel.

### The areas are given by

(Equation 3) $$Area\left(n\right)=\sum_{i=1}^{n}a_{i}$$

which is the sum of the true areas of *n* pixels *a*_*i*_ in a geographic reference coordinate system.

Table S3 shows the rules of building contour extraction based on nDSM, and Figure S5 shows the results of nDSM image extraction, with different results (yellow box represents changes) corresponding to the three different segmentation and merging scales. Compared with DSM images of real buildings in Figure S4, A_3_ in Figure S5 had the advantages of Equation 1 more obvious rectangular contours, (2) few holes inside buildings, and (3) effective elimination of scattered fragments outside the buildings by the small area removal method. Therefore, the segmentation and merging scales used in this experiment were 90 and 90, respectively.

#### Small area removal method and the morphological closure operation

The extracted building contours exhibit two shortcomings: first, roads and vegetation exhibit similar spectral feature values, leading to the formation of many small scattered fragments after vegetation removal. The morphological “Small area removal method” was used to remove the smaller misclassified areas, as shown in Equation 2. Secondly, the similarity between buildings and vegetation results in holes inside the extracted buildings and uneven building contour lines. The morphological closure operation was used to fill holes inside the buildings while smoothing the building contour, as shown in Equation 4. Figure S6 shows the results of building contour extraction.

(Equation 4) A⋅B=(A⊕B)☉B

A is the collection of extracted building surfaces, A·B is the surfaces A after closing windows of size B. ⊕ is the expansion operation, and ⊙ is a corrosion operation.

#### Segmentation of buildings through ROI

In image processing, the regions of interest (ROIs) were outlined by rectangles, circles, ellipses, irregular polygons, etc. First, the building vector contour obtained from nDSM (as shown in Figure S6B) was converted into ROI. Then, the ROI were used to extract building samples from images at different periods. Finally, fine registration was performed on images from different periods, with a minimum matching degree of 0.6 and a maximum tolerance of 5 for connection points. The buildings extracted from images at different periods through ROI are shown in Figures S7A–S7F.

#### Classification of damage types and selection of optimal window

According to *the classification of earthquake damage of buildings* (GB/T 24335-2009) (*National Standard of the People’s Republic of China*, 2009) and the relationship between remote sensing characteristics, the earthquake damage index and degree of earthquake damage of buildings (National Standard of the People’s Republic of China, 2008) were determined. The remote-sensing images of earthquake damage in the main residential areas of the disaster zone were analyzed, and the image features were used as the basis of earthquake damage interpretation by remote sensing. The earthquake damage survey shows that the seismic resistance of buildings made with adobe or brick and wood is poor and the buildings are easily destroyed. Older brick or brick-concrete buildings are also prone to damage. The seismic resistance of the reinforced concrete frame structure is slightly better. Therefore, the basis of the remote-sensing interpretation of earthquake damage depends on the type of structure and the spatial distribution characteristics of the damage, determined by remote sensing (Table S4).

Buildings are classified as “basically intact”, “moderately damaged”, and “severely damaged.” Basically intact buildings are newly built, or not substantially damaged, framed and concrete-brick structures. The overall structure of these buildings is complete, with clear external contours and no obvious damage or collapse. The earthquake damage suffered by them is relatively small, with some possibility of damage to exterior walls of these buildings. Severely damaged and moderately damaged buildings are mainly old civil and brick-and-wood structures. The load-bearing carriers of these buildings are wood, and the walls are mostly made of soil. The structure of these old buildings is simple, and their roofs are dark or black. These buildings suffered significant earthquake damage with a large areas of roof and wall collapsing. The collapsed wooden frame and some soil, tiles, and other debris can be seen. The on-site survey images of some samples are shown in Figure S8.

Because the patterns of post-earthquake buildings are complex and diverse, the selection of samples follows the rule of stable feature parameters in the same type of samples. A total of 37 samples were selected according to this principle, comprising 15 basically intact, 10 moderately damaged, and 12 severely damaged buildings. Some examples are shown in Figure S9.

Texture features are described through a grayscale co-occurrence matrix, which is related to the selected direction, step size, window size, and quantization level of images. Therefore, the parameters for generating the grayscale co-occurrence matrix need to be selected based on particular texture features of the images. Based on experience, the average of the grayscale co-occurrence matrices in the four directions of 0°, 45°, 90° and 135° was taken as the grayscale co-occurrence matrix for the central pixel position of the local image.^37^ Because the change in image quantization level has little effect on the gray level co-occurrence matrix, the default image quantization level was set to 64. The default step size for this experiment was set to 1 due to the fact that, the smaller the parameter step size, the more detailed the texture features can be reflected. The window size had the greatest impact on it. The parameter eigenvalues of three samples at different window sizes were calculated, and the window size with better discrimination of sample characteristic parameters was selected accordingly. The optimal window size was selected with mean and entropy as examples.^47^ Window sizes between 3 × 3 and 59 × 59 were used to calculate the mean and entropy of different window sizes considered in this paper, and we considered how the eigenvalues associated with building earthquake damage levels varied with the change of window size (Figure S10). It can be seen from the figure that, when the window size was 7 × 7, the mean and entropy had better discrimination for different damaged buildings. Therefore, the window size for the gray level co-occurrence matrix calculation was set to 7 × 7.

#### Texture feature change analysis and PCA

##### Texture feature change analysis

In this study, image texture features were analyzed based on GLCM. There are usually 8 parameters reflecting image texture features. If all of them are used in the calculation, information redundancy is inevitable. Selecting the optimal combination of these 8 parameters for calculation is the key point of this paper. Select 251 pixels from the damaged building sample were randomly selected in this experiment based on on-site survey images, and the characteristic values of the corresponding image pixels were statistically analyzed with a window size of 7 × 7. The combination of characteristic parameters able to best reflect the damage information of the imaged buildings was extracted. The trend change curve of different texture characteristic parameters of damaged building samples before and after the earthquake is shown in Figure S11. Some texture parameters of the damaged buildings were mixed up in the trend change curves of homogeneity (a_1_, b_1_, c_1_), contrast (a_2_, b_2_, c_2_), variance (a_5_, b_5_, c_5_), second moment (a_7_, b_7_, c_7_), correlation (a_8_, b_8_, c_8_) before and after earthquake, showing that they cannot reflect the differences in the characteristics of damaged buildings before and after earthquake (The green interval line in Figure S11 indicates the same change characteristics). The distinctions in the mean, entropy, and dissimilarity were obvious; these three characteristics can reflect well the differences in damaged buildings before and after an earthquake.

#### Image fusion

The mean, entropy, and dissimilarity have the same geographical reference and size. For this reason, the HSV (Hue, Saturation, Value) or Brovey transform is suitable. The HSV transform is suitable for stable texture and spatial features, while the Brovey transform applies to stable spectral features. In this study, the HSV image fusion mode was adopted. Figure S12 shows the fusion results for the image mean + entropy + dissimilarity for different periods.

#### PCA

After obtaining the optimal texture feature combination in the experiment, ENVI software was used to detect the PCA. As a simple, non-parametric method, PCA can suppress image noise, highlight the most important information, and improve information recognition efficiency by reducing dimensionality while maximizing the amount of information retained. The principal components contain key feature information, which can effectively solve the problem of complexity in earthquake damage identification algorithms. Reasons for using PCA in this experiment: Unlike Linear Discriminant Analysis (LDA) and Recursive Feature Elimination (RFE), PCA is an unsupervised learning method that does not rely on category labels or target variables. This allows it to reduce the dimensionality of data without prior knowledge.

#### Accuracy evaluation

In this paper, the methods of field investigation and visual interpretation of images were used to calculate the overall accuracy and kappa coefficient of 13 models respectively. Table S5 shows the confusion matrix.

(Equation 5) $$OA=\frac{a+d}{a+b+c+d}$$

(Equation 6) $$K=\frac{\left(a+b+c+d\right)\cdot \left(a+d\right)-\left[\left(a+c\right)\cdot \left(a+b\right)+\left(b+d\right)\cdot \left(b+a\right)\right]}{{\left(a+b+c+d\right)}^{2}-\left[\left(a+c\right)\cdot \left(a+b\right)+\left(b+d\right)\cdot \left(b+a\right)\right]}$$

(Equation 7) $$PA=\frac{a}{a+c}$$

(Equation 8) $$UA=\frac{a}{a+b}$$

where OA, UA, PA and K are overall accuracy, user accuracy of class ‘1’, producer accuracy of class ‘1’ and kappa coefficient, respectively.

### Quantification and statistical analysis

In this study, we used Global_mapper, ArcGIS, ENVI and eCognition to analyze the data. Liglobal_mapper extracts DOM from DSM to get nDSM. Then, eCognition and ENVI (1) were used to generate a single building image from different data sources to eliminate the influence of non-building information such as vegetation, terrain, and shadow in change detection. (2) Multi-feature fusion and principal component change detection were used to suppress information redundancy caused by multi-feature correlation and reduce noise in images. (3) Maximum Likelihood Classification (ML), Neural Net Classification (NN), Mahalanobis Distance Classification (MD), and Support Vector Machine Classification (SVM) machine learning models were selected for comparison.
