# Supplementary material for: A single-building damage detection model based on multi-feature fusion: A case study in Yangbi
Source: iScience. 2023 Nov 29;27(1):108586. doi: 10.1016/j.isci.2023.108586 (PMC10758967; doi:10.1016/j.isci.2023.108586)
Supplement: Document S1. Figures S1–S12 and Tables S1–S5 [file mmc1.pdf]

iScience, Volume *27*

Supplemental information

A single-building damage detection model based on multi-feature fusion: A case study in Yangbi

Haoguo Du, Xuchuan Lin, Jinzhong Jiang, Yongkun Lu, Haobiao Du, Fanghao Zhang, Fengyan Yu, Tao Feng, Xiaofang Wu, Guanling Peng, Shurong Deng, Shifang He, and Xianfu Bai

Supplemental Figures

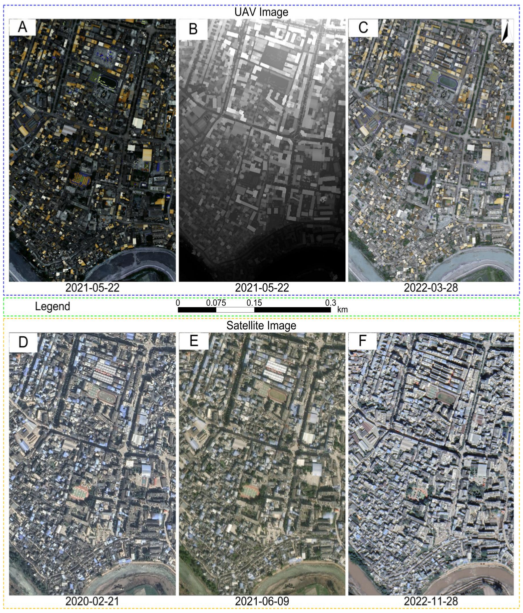

Figure S1. Image data of the study area. (A) UAV DOM image on May 22, 2021; (B) UAV DSM image on May 22, 2021; (C) UAV DOM image on March 28, 2022; (D) Satellite DOM image on February 21,2020; (E) Satellite DOM image on June 9, 2021;(F) Satellite DOM image on November 28, 2022. Related to Figure 1.

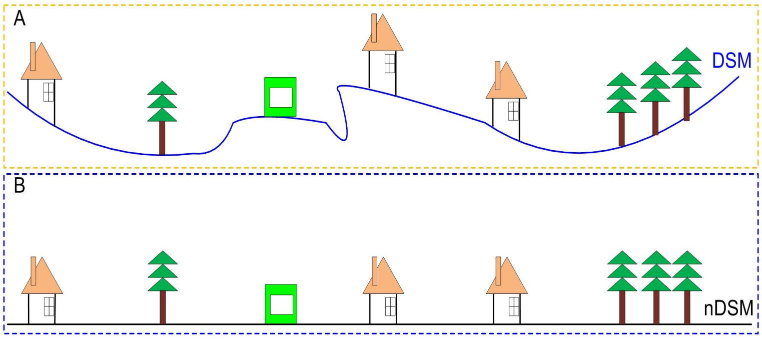

Figure S2. DSM and nDSM schematic. (A) Terrain + object path profile in DSM image; (B) Terrain + object path profile in nDSM image. Related to Figure 3.

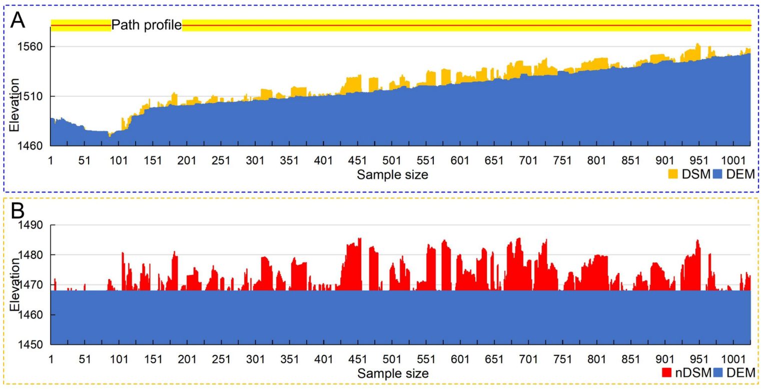

Figure S3. DSM, DEM and nDSM path profile results. (A) Digitization result of terrain + object path section in DSM+DEM image; (B) Digitization result of terrain + object path section in nDSM+DEM image. Related to Figure 3.

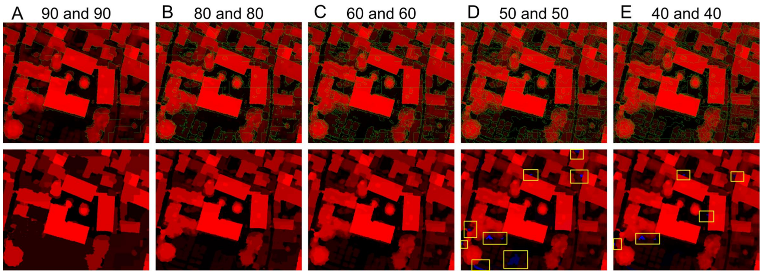

Figure S4. Experimental results of segmentation and merging of real objects in DSM images. (A) Experimental results of the image segmentation scale and merging scale parameters are all 90; (B) Experimental results of the image segmentation scale and merging scale parameters are all 80; (C) Experimental results of the image segmentation scale and merging scale parameters are all 60; (D) Experimental results of the image segmentation scale and merging scale parameters are all 50; (E) Experimental results of the image segmentation scale and merging scale parameters are all 40. Related to Figure 9.

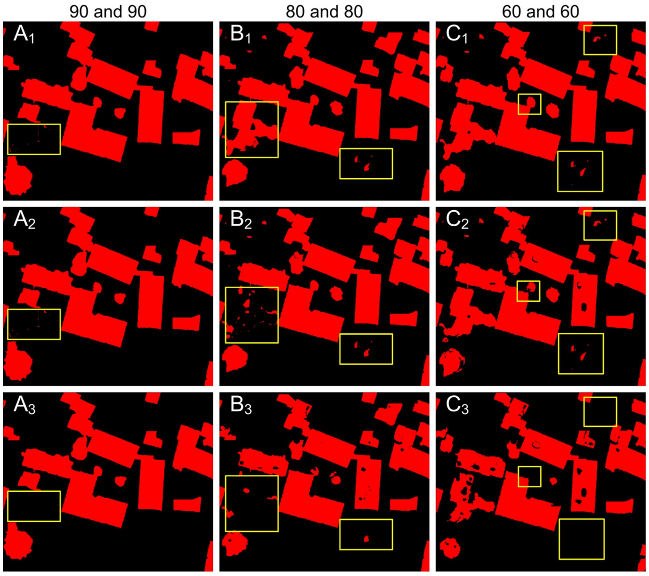

Figure S5. The results of nDSM image extraction. The segmentation and merging scale of nDSM were both 90: (A_1) (169.907＜*AvgBand* 1＜253.064), (A_2) (169.907＜*AvgBand* 1＜253.064 and 0.223＜*Rect_fit*＜1), (A_3) (169.907＜*AvgBand* 1＜253.064 and 0.223＜*Rect_fit*＜1 and 400＜*Area*＜669166); The segmentation and merging scale of nDSM were both 80: (B_1) (169.907＜*AvgBand* 1＜253.538), (B_2) (169.907＜*AvgBand* 1＜253.538 and 0.411＜*Rect_fit*＜1), (B_3) (169.907＜*AvgBand* 1＜253.538 and 0.411＜*Rect_fit*＜1 and 1000＜*Area*＜173986); The segmentation and merging scale of nDSM were both 60: (C_1) (169.907＜*AvgBand* 1＜253.538), (C_2) (169.907＜*AvgBand* 1＜253.538 and 0.4＜*Rect_fit*＜1), (C_3) (169.907＜*AvgBand* 1＜253.538 and 0.4＜*Rect_fit*＜1 and 2000＜*Area*＜136025). Related to Table S3 and Figure 9.

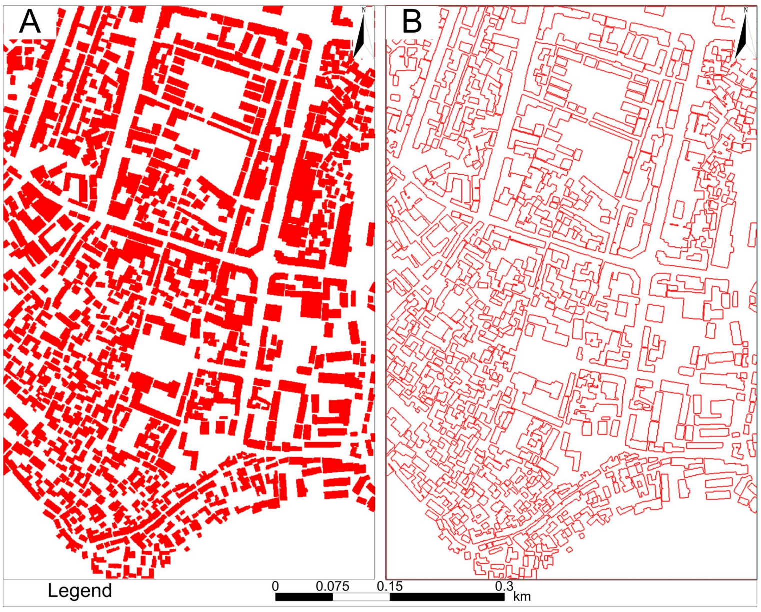

Figure S6. Building contour extraction. (A) Binary graph; (B) Results of building contour extraction. Related to Figure 4 and Figure 5.

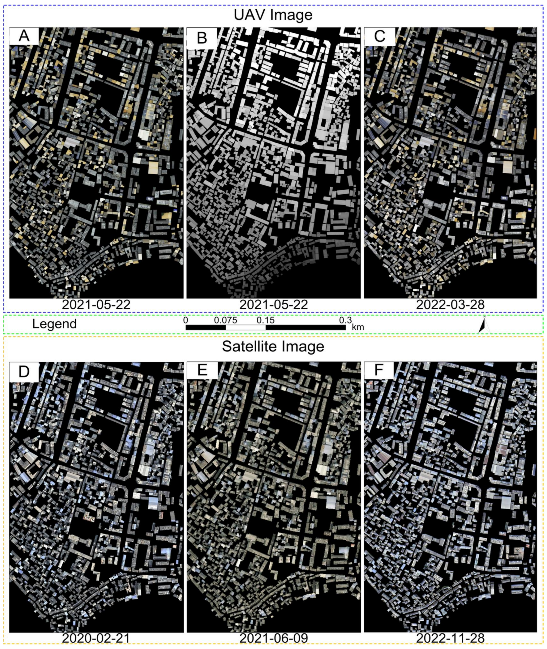

Figure S7. Extraction results for a single building. (A) UAV DOM image from May 22, 2021; (B) UAV DSM image from May 22, 2021; (C) UAV DOM image from March 28, 2022; (D) Satellite DOM image from February 21, 2020; (E) Satellite DOM image from June 9, 2021; (F) Satellite DOM image from November 28, 2022. Related to Figure 4 and Figure 5.

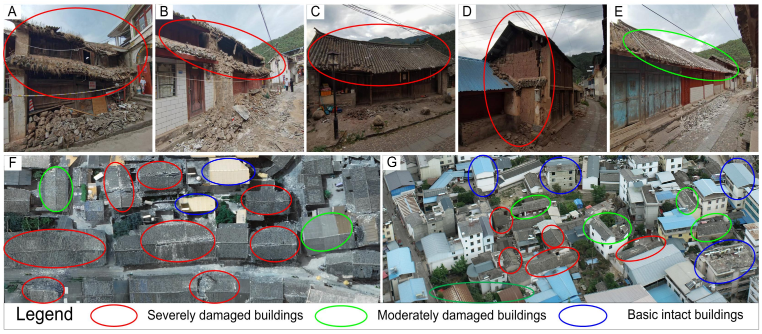

Figure S8. The slanted image obtained after an earthquake in Yunlongqiao town in Yangbi County. Field survey image: (A) Field research Severely Damaged buildings sample 1; (B) Field research Severely Damaged buildings sample 2; (C) Field research Severely Damaged buildings sample 3; (D) Field research Severely Damaged buildings sample 4; (E) Field research Moderately Damaged buildings sample 1; (F) UAV is photographing DOM and building destruction classification;(G) UAV tilting DOM image and building destruction classification. Related to Figure 5.

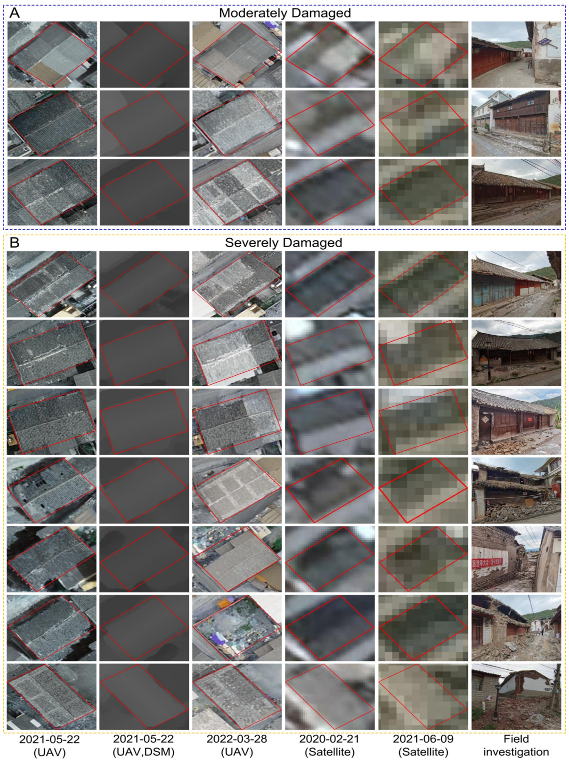

Figure S9. Damaged building sample. (A) Field research Moderately Damaged buildings sample of a positive photograph; (B) Field research Severely Damaged buildings sample of a positive photograph. Related to Figure 1.

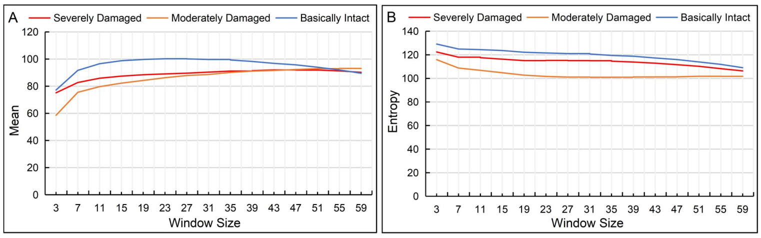

Figure S10. Texture feature parameter changes curve with window size. (A) The mean varies with the window size; (B) The entropy varies with window size. Related to Figure 4.

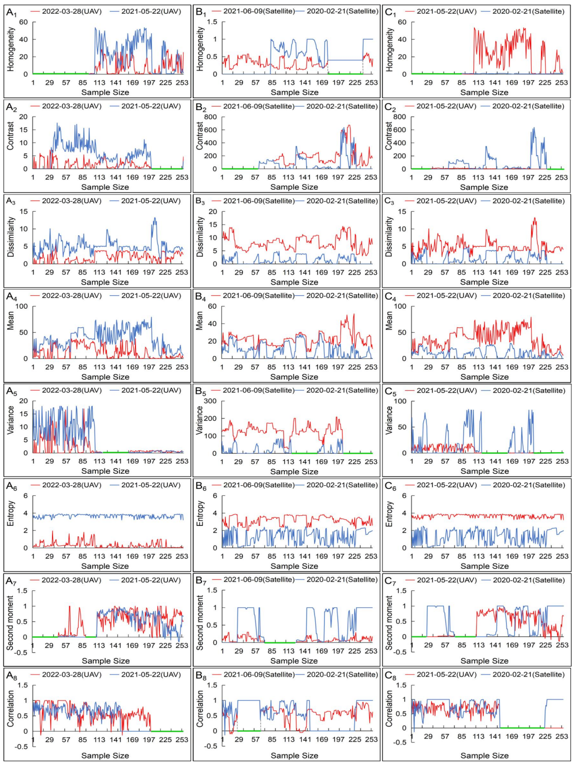

Figure S11. The trend change curves of different texture characteristic parameters of damaged buildings samples before and after earthquake. Homogeneity (A_1, B_1, C_1); Contrast (A_2, B_2, C_2); Dissimilarity (A_3, B_3, C_3); Mean (A_4, B_4, C_4); Variance (A_5, B_5, C_5); Entropy (A_6, B_6, C_6); Second moment (A_7, B_7, C_7); Correlation (A_8, B_8, C_8). Related to Figure 4 and Figure 11.

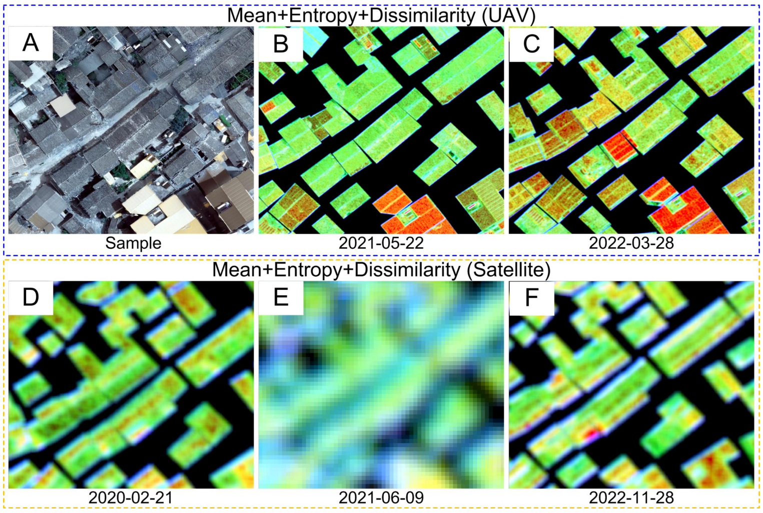

Figure S12. Fusion results of image mean + entropy + dissimilarity in different periods. (A) Sample; (B) The result of mean + entropy + dissimilarity fusion in UAV DOM image from May 22, 2021; (C) The result of mean + entropy + dissimilarity fusion in UAV DOM image from March 28, 2022; (D) The result of mean + entropy + dissimilarity fusion in Satellite DOM image from February 21, 2020; (E) The result of mean + entropy + dissimilarity fusion in Satellite DOM image from June 9, 2021; (F) The result of mean + entropy + dissimilarity fusion in Satellite DOM image from November 28, 2022. Related to Figure 4 and Figure 11.

Supplemental Tables

Table S1. Image information. Related to Figure 1 and Figure S1.

Fig.S1	SO	TY	DA	SI(MB)	RE(m)	CO(pixels)	RO(pixels)	FO	BA	PCS
(A)	UAV	DOM	2021-05-22	698	0.045	12494	19521	TIFF	3	WGS_1984_UTM_Zone_47N
(B)		DSM	2021-05-22	170	0.091	6179	9653			
(C)		DOM	2022-03-28	392	0.06	9370	14640			
(D)	Google Satellite	DOM	2020-02-21	18.2	0.285	2134	2969			
(E)		DOM	2021-06-09	1.13	1.14	528	739			
(F)		DOM	2022-11-28	18.2	0.285	2134	2969			

SO, Source; TY, Type; DA, Data; SI, Size; RE, Resolution; CO, Column; RO, Row; FO,Format; BA, Bands; PCS, Projection Coordinate System.

Table S2. Image acquisition device parameters. Related to Figure 1.

Aircraft Performance	Technical Parameters	Aircraft Performance	Technical Parameters
Model Number	Phantom 4 Pro	Number of Flights	8 times
Total Weight	1388 g	Image course overlap rate	70%
Flight altitude	200 ± 5m	Image lateral overlap rate	65%
Single Flight Time	13 ± 2min	Total Image Area	0.6964 km^2
Hover Accuracy	Vertical Direction ± 0.1m<br>Horizontal Direction ± 0.1m	Image Format	TIFF (DOM)<br>TIFF (DSM)
Angle of Dip	-90°	Image count	12840 Sheets
Flight Speed	28.8 km/h	Automatic Photo Mode	Obtain Images at Intervals
Single Image Size	4864×3648	Lens	FOV 84° 8.8 mm/24 mm<br>With Auto Focus

Table S3. Extraction of building contour rules based on nDSM. Related to Figure S5 and Figure 9.

Split and merge scales	Rule	Figure S5
90 and 90	if	
	169.907＜*AvgBand* 1＜253.064	A_1
	and 0.223＜*Rect_fit*＜1	A_2
	and 400＜*Area*＜669166	A_3
80 and 80	if	
	169.907＜*AvgBand* 1＜253.538	B_1
	and 0.411＜*Rect_fit*＜1	B_2
	and 1000＜*Area*＜173986	B_3
60 and 60	if	
	169.907＜*AvgBand* 1＜253.646	C_1
	and 0.4＜*Rect_fit*＜1	C_2
	and 2000＜*Area*＜136025	C_3

Table S4. Damage characters from remote sensing images by Yangbi M_S 6.4 earthquake. Related to Table 1.

Degree of damage	Image features	Actual degree of damage by the earthquake
Severely damaged	The outline of the building in the image can be differentiated but most (40%–70%) of it is unclear. By contrast, the layout is fairly clear. The shadow disappears, and the debris accumulation resulting from the collapse or partial collapse is shown. The tone of the ruins is light, the texture is not uniform, the range varies greatly, and the local distribution is fragmented. The texture and tone of the post-earthquake image are substantially different from those of the same sensor before the earthquake.	The severely damaged buildings are mainly old adobe and brick and wood structures in which the load-bearing carrier is wood, the walls are mostly made of soil, the roof is black, the structure is simple, and the construction time is long. The buildings were damaged by the earthquake and large areas of the roof and wall collapsed. The collapse of the wooden frame and some soil, tiles, and other debris are visible. Collapse of the roof or large holes cause substantial changes in the texture features. These buildings cannot be repaired and should be rebuilt.
Moderately damaged	The single house is easy to identify in the image, and the spatial layout of the group of houses is clear, but the outline of a few houses (10%–45%) is unclear, showing the accumulation of debris formed by destroyed roofs. The ruins are light in tone, uneven in texture, and limited in distribution.	The moderately damaged houses were mainly brick and wood structures and brick and concrete structures. The roofs were all tiles, and some tiles fell off after the earthquake, but the overall structure was not affected as seen in the image. The damage resulted in slight changes to the texture features. After repair, the building can be inhabited.
Basically intact	In the image, the outline of the house is clear and its shape is regular. The layout of the group of houses is clear and complete with no visible speckle image features of debris accumulation. Scattering (<10%) is only seen in the accumulation of debris formed by the collapse of the house. Local collapse can be seen in the texture image features.	Undamaged and slightly damaged houses were mainly structures made with modern frames and brick and concrete. The overall structure of these houses is complete, the external outline is clear, and there is no obvious damage or collapse. The earthquake damage is thus small, with some external walls showing slight scarring; minor damage to frames, and no damage to the roof. The building can be used immediately.

Table S5. Sample confusion matrix generated for two classes Related to Table 4 and Table 5.

	**Reference**	
	Class'1'	Class'2'
Class'1'	*a*	*b*
Class'2'	*c*	*d*
